# MetE: a promising protective antigen for tuberculosis vaccine development

**DOI:** 10.3389/fimmu.2025.1593263

**Published:** 2025-07-21

**Authors:** Salem Salman Almujri, Elena Stylianou, Annalisa Nicastri, Iman Satti, Marcellus Korompis, Shuailin Li, Christopher J. De Voss, Marco Polo Peralta Alvarez, Rachel Tanner, Paulo J. G. Bettencourt, Nicola Ternette, Helen McShane

**Affiliations:** ^1^ The Jenner Institute, University of Oxford, Oxford, United Kingdom; ^2^ Department of Pharmacology, College of Pharmacy, King Khalid University, Asir-Abha, Saudi Arabia; ^3^ Chester Beatty Laboratories, The Institute Cancer Research, London, United Kingdom; ^4^ Department of Biology, University of Oxford, Oxford, United Kingdom; ^5^ Universidade Católica Portuguesa, Faculty of Medicine, Center for Interdisciplinary Research in Health, Lisbon, Portugal; ^6^ School of Life Sciences, University of Dundee, Dundee, United Kingdom

**Keywords:** tuberculosis, mycobacterium tuberculosis, vaccines, immunopeptidomics, mass spectrometry, immunoinformatics, antigen discovery, HLA/MHC

## Abstract

**Introduction:**

Tuberculosis (TB), caused by *Mycobacterium tuberculosis* (MTB), remains a significant global health concern. The existing vaccine, Bacillus Calmette-Guérin (BCG), provides inconsistent protection, highlighting the pressing need for a more effective vaccine. We aimed to identify novel *MTB* antigens and assess their protective efficacy as TB vaccine candidates.

**Methods:**

Using immunopeptidomics, we identified 64 and 80 unique mycobacterial antigens derived from BCG and MTB, respectively. We prioritised antigens based on HLA allele coverage through an immunoinformatics approach.

**Results:**

The candidates, *hisD*, *metE*, and *mmpL12*, delivered as DNA vaccines, were evaluated for efficacy in mice using the ex vivo Mycobacterial Growth Inhibition Assay (MGIA) and *metE* was identified as a promising candidate. In vivo murine *MTB* challenge experiments confirmed the protective efficacy conferred by *metE* when formulated as recombinant protein with AS01™ or AddaS03™ adjuvants, compared to the naïve group. The immunogenic profiles of *metE* formulated in the two different adjuvants differed, with *metE*-AS01™ inducing antigen-specific IFN-γ, TNF-α, IL-2, IL-17, IgG1 and IgG2a-c, while *metE*-AddaS03™ induced TNF-α, IL-2, IL-17, IL-4, IgM, IgG1, IgG2b.

**Conclusion:**

Our findings highlight *metE* as a promising protective antigen for future TB vaccine development.

## 1 Introduction

Tuberculosis (TB) remains a significant global health challenge despite being both preventable and treatable. According to the recent Global TB Report by the World Health Organisation, TB has regained its position as the leading cause of death from a single infectious agent, surpassing COVID-19. The causative agent, *Mycobacterium tuberculosis* (*MTB*), infected 10.8 million people and caused 1.25 million deaths in 2023 (1). Bacillus Calmette-Guérin (BCG), comprised of live attenuated *Mycobacterium bovis*, is the only licensed vaccine for TB, but has little protective effect against pulmonary disease in adolescents and adults (2, 3). There is an urgent need for the development of more effective preventive strategies.

The importance of cell-mediated immunity (CMI) in controlling TB, primarily through a T-helper 1 CD4+ T-cell response, has been demonstrated in HIV-infected individuals. These individuals experience a decrease in the number of CD4+ T-cells, making them highly susceptible to *MTB* infection and reactivation of latent *MTB* infection (LTBI) (4). In addition, accumulating evidence indicates that T-helper 17 (Th17) CD4^+^ T cells contribute to protective immunity against MTB (5). There is also evidence for a role for CD8+ T cells (6, 7), and emerging evidence that antibodies may play a role in immunity to TB (8). To present *MTB*-derived antigens to T cells, host immune cells such as macrophages and dendritic cells use major histocompatibility complex (MHC) molecules (9). CD4+ T cells recognize peptides presented by MHC class II (MHC-II) molecules, while CD8+ T cells recognize peptides presented by MHC class I (MHC-I) molecules (10). These interactions between T cells and MHC molecules are critical for activating and shaping the adaptive immune response to *MTB* infection. Since no single T cell subset has been definitively correlated with protection, inducing a diverse CD4^+^ T cell response remains a key objective in TB vaccine development, supported by strong and consistent evidence of CD4^+^ T cell-mediated protection in both preclinical and clinical studies (11–13). In contrast, CD8^+^ T cell responses have been more variable, and clinical evaluations of TB vaccine candidates have shown that CD8+ T cell responses were relatively poorly induced when compared to CD4+ T cell responses (14).

The discovery of novel protective antigens is key to the development of candidate subunit vaccines aimed at improving BCG efficacy. Subunit vaccines, composed of one or a few mycobacterial antigens delivered with a platform of choice, have better safety profiles than live attenuated vaccines (15). However, with over 4000 genes (16), the identification of mycobacterial antigens presented on MHC molecules has been challenging (17, 18). Immunopeptidomics, a mass spectrometry-based approach, is one of the methods used to identify potentially immunogenic mycobacterial antigens by analyzing peptides presented by MHC molecules in infected cells (19). By identifying pathogen peptides and their corresponding precursor antigens, it is possible to infer which proteins are processed and presented to the immune system and which are more likely to elicit a T-cell response. Since the 1990s, numerous research groups have applied immunopeptidomic techniques to discover novel antigens presented by MHC molecules in the context of cancers (20, 21) and infectious diseases including TB (22, 23). A study conducted by Flyer et al. identified three peptides associated with MHC-I, all derived from a single antigen (Rv0341), after infecting the U937 histiocytic lymphoma cell line with *MTB* (24). Similarly, another study identified 16 *MTB* antigens linked to MHC-I in primary human macrophages sourced from six donors with LTBI (25). Targeting non-classical MHC-I molecules also yielded 28 unique antigens (26). Recently, our group identified 94 mycobacterial peptides presented by MHC-II and 43 presented by MHC-I, in BCG-infected THP-1 cells, using immunopeptidomics. From these, three antigens [galactofuranosyl transferase (glfT2), isoniazid inductible gene (iniB) and probable fatty acid synthase (fas)] were expressed in viral vectors and evaluated as vaccine candidates in a murine aerosol *MTB* challenge model. When delivered as a boost to previous BCG vaccination, the combination of these vectors conferred significant protection in mice, compared to BCG alone (27).

To accelerate TB vaccine development, we employed a mass spectrometry-based immunopeptidomics approach to identify novel mycobacterial antigens. Analysis of peptides presented on MHC molecules in human macrophages (differentiated THP-1 cells) infected with either BCG or *MTB* led to the identification of 64 BCG-derived and 80 *MTB*-derived antigens. Because those identified antigens resulted from specific HLA alleles in THP-1 cells, we expanded the screening of T cell epitopes using the entire antigen sequence against the most frequent HLA alleles worldwide to maximize population coverage, which is important for vaccine development. To address this, immunoinformatic tools such as NetMHCpan and NetMHCIIpan were utilized to map T cell epitopes within a given antigen sequence (28). These tools were employed to rank antigens identified from immunopeptidomics data based on their T cell epitope richness. By predicting T cell epitopes against the most prevalent MHC alleles globally (29, 30), the development of vaccines capable of eliciting robust T-cell responses across diverse populations. Although our primary focus was on T cell epitope analysis, using the full-length antigenic sequence preserved the structural integrity of the antigens. This in turn, allowed for the potential induction of antibodies that recognize native conformations, thereby indirectly accounting for antibody responses as part of our overall vaccine design strategy.

A prioritization pipeline of identified antigens using immunopeptidomic and immunoinformatic tools highlighted candidate antigens for further assessment. From this list, three top candidates were selected for evaluation in mice using the *ex vivo* mycobacterial growth inhibition assay (MGIA), with one antigen, metE, emerging as a promising candidate due to its strong immunogenic profile. In murine *MTB*-challenge experiments, metE provided protection when administered as a recombinant protein formulated with either AS01™ or AddaS03™ adjuvants, with each formulation inducing distinct immune responses. These adjuvants were chosen for their clinical relevance and proven ability to elicit broad immune responses, including diverse effector T cell subsets and functional antibodies across multiple species (31, 32). AddaS03™ shares compositional similarities with AS03^®^, an adjuvant used in licensed influenza vaccines (33), while AS01™ is a component of the Shingrix vaccine (AS01B) and has demonstrated efficacy against TB in the M72/AS01E vaccine candidate (34). By using adjuvants with proven translational potential, minimizes the risk of generating protective responses in preclinical models that may not be reproducible in humans. Our findings indicate that metE is a promising novel antigen for inclusion in a subunit vaccine against TB.2 Results

## 2 Results

### 2.1 Identification and selection of mycobacterial antigens

**Figure 1:**
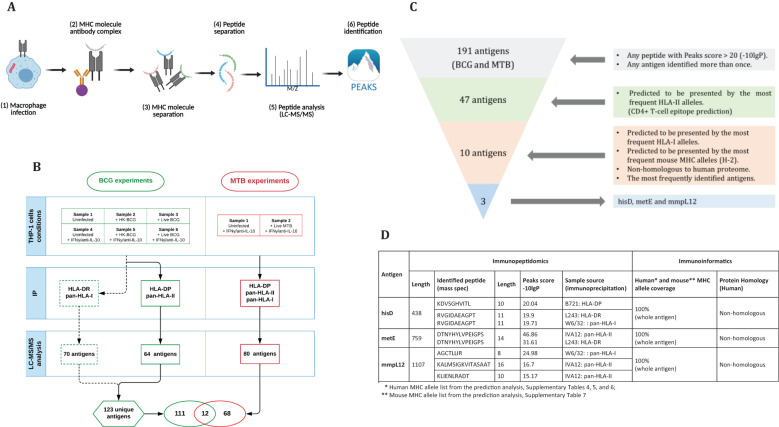
**(A)** Step-by-step procedure for antigen identification: THP-1 macrophages are infected with mycobacteria, harvested, lysed for immunoprecipitation, and peptides are eluted from MHC complexes (generated by BioRender). The peptides are separated by HPLC, analyzed via LC-MS/MS, and processed using Peaks software (v10). **(B)** Sample arrangement for immunopeptidomics analysis: In the BCG experiments, Bettencourt et al. (2020) prepared six samples, which underwent IP with L243 and W6/32 antibodies, identifying 70 antigens. Additional IP with B271 and IVA12 isolated HLA-DP and -DQ, identifying 64 antigens. In the MTB experiments, two samples underwent IP with three antibodies, leading to the identification of 80 antigens, totally 191 unique antigens. **(C)** Antigen selection criteria: selecting antigens with a Peaks score >20 or identified multiple times yielded 47 antigens out of 191. Antigens with full HLA-II coverage were narrowed down to 10. Finally, non-homologous antigens presented by common HLA-I alleles resulted in three candidates: hisD, metE, and mmpL12. **(D)** Characterization of candidate antigens using immunopeptidomics and immunoinformatics.

Building on the work of Bettencourt et al. (27) which identified HLA-I and HLA-DR peptides from THP-1 cells infected with BCG using W6/32 and L243 antibodies respectively, resulting in the identification of 70 antigens (**Figures 1A, B**, dotted lines), remaining HLA molecules were investigated, specifically HLA-DP and HLA-DQ, using the same samples (**Figures 1B**, samples 1-6). To achieve this, antibodies targeting HLA-DP (B721), followed by a pan-HLA-II antibody (IVA12, non-selective HLA-II antibody) were employed to enrich for HLA-DQ and any remaining HLA-II molecules, particularly HLA-DR. This approach identified 39 BCG peptides associated with HLA-DP and 34 peptides associated with other class II molecules, resulting in a total of 64 unique antigens (**Figure 1B**; **Supplementary Table 1**). Interestingly, the number of identified peptides increased in samples treated with IFN-γ (**Supplementary Figures 1C, D**).

Subsequently, a second set of experiments was conducted using *MTB* as the infectious agent. Two samples were prepared under two conditions (**Figures 1A, B**) and subjected to sequential immunoprecipitation with antibodies specific for HLA-DP (B721), pan-HLA-II (IVA12) to enrich for HLA-DQ and HLA-DR, and pan-HLA-I (W6/32). This workflow identified 87 peptides derived from 80 unique *MTB* antigens (**Figure 1B**; **Supplementary Table 2**).

The immunopeptidomics analysis revealed distinct peptide length distributions for the two HLA classes. Peptides presented by MHC-I molecules were predominantly 8 to 10 amino acids long, consistent with the expected size for MHC-I-presented peptides; peptides presented by MHC-II molecules were primarily between 13 and 17 amino acids, characteristic of MHC-II-presented peptides (**Supplementary Figure 2**). Shorter peptides were also observed in the MHC-II immunoprecipitates, likely due to co-immunoprecipitation of MHC-I peptides and the intrinsic ability of MHC-II molecules to accommodate peptides of varying lengths, including shorter ones (35).

By combining the identified mycobacterial antigens from BCG (123 antigens, including those reported by Bettencourt et al. (27) and *MTB* (80 antigens), we assembled a dataset of 191 unique antigens, 12 of which were shared between the two pathogens (**Figures 1B, C**; **Table 1**). Given the substantial number of identified antigens, we developed a streamlined *in silico* approach to prioritize and select candidate antigens. This approach integrated immunopeptidomic and immunoinformatic parameters and involved a three-step selection process (**Figures 1C**). First, antigens were ranked based on their Peaks score, which reflects the accuracy of sequence identification. Second, MHC allele coverage was evaluated using NetMHCpan and NetMHCIIpan tools to map T-cell epitope-rich antigens against the most prevalent HLA alleles, ensuring broad immunogenic potential. Finally, homology between mycobacterial antigens and the human proteome was assessed using the Vaxign server. This server employs BLAST to identify sequence similarities, and proteins with an Expect value (E-value) less than 1 × 10^5^ were considered homologous (36). Consequently, antigens exhibiting an E-value below this threshold were excluded to avoid potential risk of cross-reactive immune responses against self-antigens (37, 38). Through this rigorous selection process, we identified three top-ranked candidate antigens: hisD, metE, and mmpL12. These antigens demonstrated broad HLA coverage for both class I and II alleles, and exhibited low homology with the human proteome (**Figure 1D**; **Supplementary Table 2**).

**Table 1:** Identified BCG and MTB peptides of shared antigens.

No	BCG peptides	BCG-*MTB* shared antigens	*MTB* peptides
**1**	PVKAKLAPVP	Rv0426c (BCG_0465c)	ALAIGAI
**2**	KPADGVPPPPLNTKLPEDPPP	Rv2164c (BCG_2181c)	ARAKARKAKAPK RAKARKAKAPK
**3**	KPNPIGVGLME	Rv3239c (BCG_3788)	LENVRPLA
**4**	APLLAELIRGGAALSRVRHPGD	Rv3776 (BCG_3838)	IGPTHTL
**5**	**NGEEYLILSARDVL**A **YLILSARDVLA** **YLILSARDVLA** **NGEEYLILSARDVL** **NGEEYLILSARDVL** IKYNGEEYLIL	groS Rv3418c (BCG_3488c)	**NGEEYLILSARDVL**A **NGEEYLILSARDVL**
**6**	**RVGIDAEAGPT** KDVSGHVITL	hisD Rv1599 (BCG_1637)	**RVGIDAEAGPT**
**7**	KPLVRHTVHT	nrp Rv0101 (BCG_0134)	APGAVDPAGLRAQLAQRLPGYLVP
**8**	GGAGGAGGWLIGQSGSTGGGGAGG	PE_PGRS48 Rv2853 (BCG_2873)	GTGGAGGAGGLGGHGGAGGLLIGNG
**9**	GTGGNASATGT	PE_PGRS53 Rv3507 (BCG_3571)	QALTAGAGAYAFAEAA
**10**	**TTYTLEYDG**	PE1 Rv0151c (BCG_0187c)	**TTYTLEYDG**
**11**	GPAFAALS	ppsD Rv2934 (BCG_2956)	GFAEIAL
**12**	**AQTGVYEDLLAAGVADPVK** LQDMAILTGGQVIS TGVYEDLLAAGVADPVK	groEL2 Rv0440 (BCG_0479)	**AQTGVYEDLLAAGVADPVK** PLKQIAFNSGLEPGVVAEKVRNLP DMAILTGGQVISEE DMAILTGGQVISEEVG

Bold indicates similar sequence(s).

### 2.2 Confirmation of candidate antigen expression in DNA constructs

**Figure 2:**
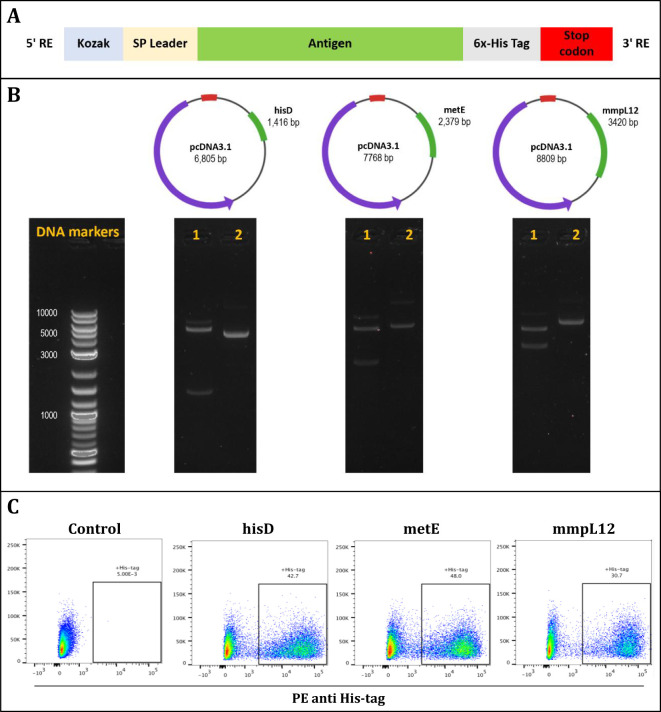
The design and validation of antigen inserts (hisD, metE, and mmpL12). **(A)** Schematic representation of the plasmid constructs showing insertion of Kozak sequences and signal peptides at the N-terminus, and a 6x-His tag followed by a stop codon at the C-terminus of each insert. **(B)** DNA agarose gels confirming successful plasmid assembly for hisD, metE, and mmpL12 constructs; lane 1 shows restriction enzyme-digested plasmids with bands at expected insert sizes, lane 2 shows uncut plasmids, and DNA markers are provided as size references. **(C)** Flow cytometry analysis confirming protein expression of each antigen (hisD, metE, mmpL12) following transfection of HEK293T cells, detected using an anti-6x-His antibody.

**Figure 3:**
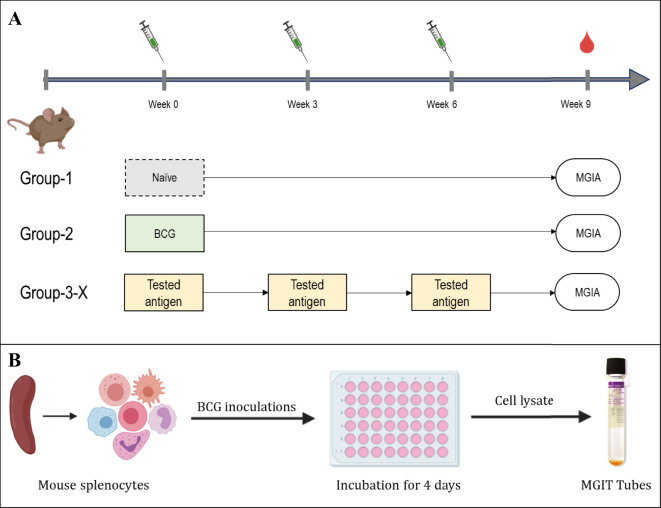
Experimental layout of MGIA experiments. **(A)** immunisation layout. CB6F1 mice were randomly divided into six groups, each containing eight mice. A single group was given a one-time intradermal BCG vaccination, while four groups designated for antigen testing received plasmid DNA vaccinations three times, with two-week intervals. One group of animals remained unvaccinated. **(B)** Schematic of the MGIA assay procedure; Splenocytes are co-cultured with approximately 300 or 500 CFU of BCG. After a 96-hour incubation period, cells were lysed, and both extracellular and intracellular bacteria were introduced to the BACTEC MGIT system and monitored until the time to detection (TTD) registered as positive.

To evaluate the protective efficacy of the three candidate antigens, DNA constructs encoding each antigen were individually generated (**Figure 2**). Mice were immunized three times with the DNA constructs at two-week intervals. Two weeks following the final immunization, we employed the *ex vivo* Mycobacterial Growth Inhibition Assay (MGIA) as a surrogate of protective efficacy (39). The MGIA was performed using splenocytes collected from vaccinated and control animals to measure mycobacterial growth inhibition, as measured by Time to Detection (TTD) (**Figure 3**).

**Figure 4:**
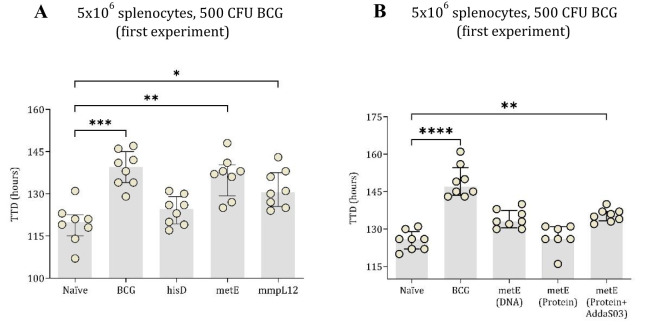
TTD values of mycobacterial growth as measured by MGIA. In the first experiment, mouse splenocytes, counted at 5x10^6^ **(A)**, were co-cultured with 500 CFU of BCG. These splenocytes were taken from both unvaccinated (naïve) mice and those vaccinated with BCG or DNA vaccines expressing hisD, metE or mmpL12. In the second experimental setup, TTD values were derived from 5x10^6^ mouse splenocytes co-cultured with BCG, inoculated at ~500 CFU **(B)**. Splenocytes originated from naïve mice and those vaccinated with BCG, metE, metE-AddaS03™, or metE-DNA. Plotted circles represent individual mice, lines representing median data, and whiskers indicating interquartile ranges (IQR). For identifying significant deviations compared to the naïve control, a combination of the Kruskal-Wallis and Dunn’s multiple comparisons tests was applied. Significance levels are denoted as * for P values <0.05, ** for P values <0.01, and *** indicating P values <0.001.

Spleen cells (5 × 10^6^ cells/well) were co-cultured with ~500 CFU of BCG. Splenocytes from mice vaccinated with BCG, metE DNA, or mmpL12 DNA showed statistically significant inhibition of mycobacterial growth compared to those from naïve mice (**Figures 4A, B**). The BCG group had the highest TTD (p = 0.0002), followed by the metE (p = 0.0017) and mmpL12 (p = 0.045) DNA vaccine groups. No significant inhibition of mycobacterial growth was noted in groups vaccinated with hisD (**Figure 4A**).

To further investigate the protective potential of the metE antigen, its efficacy as a protein-adjuvant vaccine was evaluated using the MGIA. In this experiment, 5 × 10^6^ splenocytes were co-cultured with BCG at 500 CFU. Both the BCG-vaccinated group and the metE-AddaS03™ protein-adjuvant group significantly inhibited mycobacterial growth compared to the unvaccinated group (p = 0.0001 and p = 0.01, respectively; **Figure 4B**). The metE DNA vaccine group did not exhibit significant bacterial inhibition at 500 CFU (p = 0.0604; **Figure 4B**) but provided protection at BCG inoculum of 300 CFU (p = 0.0311; **Supplementary Figure 3**).

### 2.3 Assessment of metE antigen immunogenicity and efficacy using *in vivo* aerosol *MTB* challenge studies

**Figure 5:**
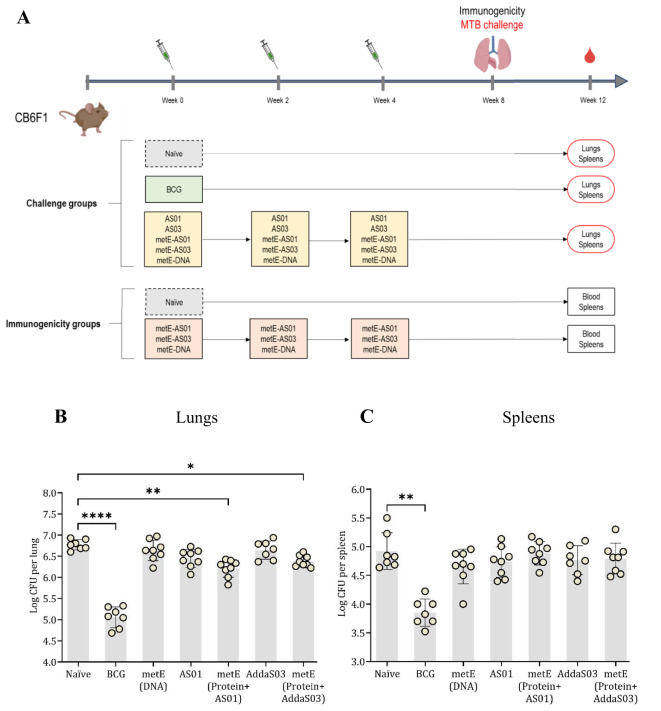
Aerosol MTB-Challenge and Immunogenicity Experiments. **(A)** MTB challenge, immunisation schedule and experimental groups. **(B)** lung and **(C)** spleen CFU counts. Plotted circles represent individual mice, lines representing median data, and whiskers indicating interquartile ranges (IQR). For identifying significant deviations compared to the naïve control, a combination of the Kruskal-Wallis and Dunn’s multiple comparisons tests was applied. Significance levels are denoted as * for P values <0.05, ** for P values <0.01, and **** indicating P values <0.0001.

Following promising results from the MGIA experiments, we then assessed whether the metE antigen, when delivered as a DNA vaccine or as a protein/adjuvant combination, could protect against an aerosol *MTB* challenge in mice (**Figure 5A**). The metE protein was formulated in two distinct adjuvants, AddaS03™ and AS01™, capable of inducing robust immune responses with a high safety profile (31, 40). Given the absence of definitive immune correlates of protection from TB, both formulations were evaluated. Additionally, recent clinical trials have demonstrated the promising efficacy of AS01™ in a candidate subunit TB vaccine (34), prompting us to assess the performance of metE combined with this adjuvant. Vaccinated and control animals were challenged with aerosolized *MTB* and lungs and spleens were collected for bacterial enumeration at the end of the study (**Figure 5A**).

Vaccination with metE administered as a protein with either adjuvant resulted in a statistically significant reduction in bacterial burden in the lungs compared to the unvaccinated groups (metE + AS01™, p = 0.0027; metE + AddaS03™, p = 0.0381). No protection was observed in the lungs when metE was administered as a DNA vaccine. In the spleen, vaccination with metE, whether administered as DNA or protein with adjuvant, did not significantly reduce bacterial load (**Figures 5B, C**). The BCG-vaccinated control group had a significant reduction in bacterial load in both lungs and spleen as expected (p = 0.0001 and p = 0.0017, respectively; **Figures 5B. C**).

**Figure 6:**
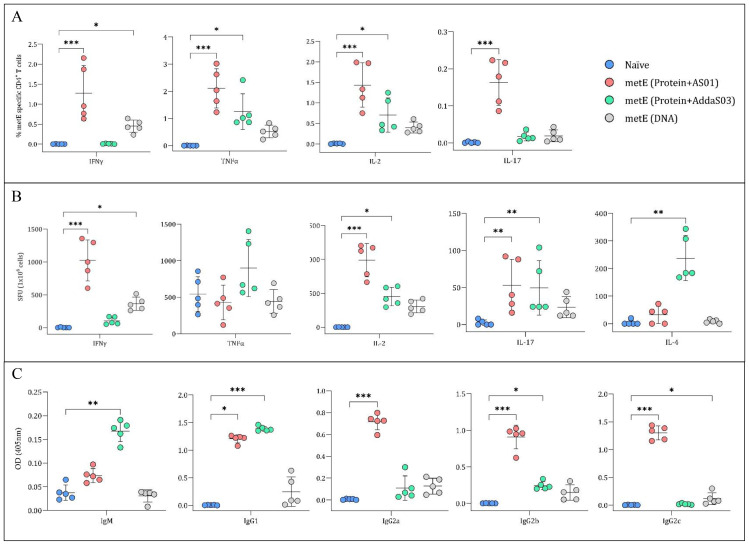
Analysis of Cytokine Secretion and metE-specific Antibody Titres. **(A)** Percentage of CD4+ T cells producing different cytokines. Mouse splenocytes were stained for CD4+ surface markers and cytokines (IFNγ, TNFα, IL-2, and IL-17) after overnight stimulation with metE protein. Cytokine staining was analysed by flow cytometry. **(B)** Cytokine secretion measured by ELISpot. Splenocytes were stimulated overnight with metE protein to measure the release of IFNγ, TNFα, IL-2, IL-4, and IL-17 cytokines. **(C)** MetE-specific antibody titers. Antibody titers (IgM, IgG1, IgG2a, IgG2b, and IgG2c) were analysed from mouse sera collected after the final vaccination by ELISA. Each plotted circle represents individual mice, with lines denoting median values. Significant differences compared to the naïve control were identified using the Kruskal-Wallis test with Dunn’s multiple comparisons test. Significance markers: * for P values <0.05, ** for P values <0.01, and *** for P values <0.001.

Immunogenicity induced by the different formulations of metE antigen was also evaluated. Each formulation induced distinct humoral and cellular immune responses, reflecting a bias towards different T helper cell profiles (**Figures 6A-C**). MetE protein formulated with AS01™ adjuvant generated a robust Th1-biased CD4+ T cell response, characterized by antigen-specific IFN-γ, TNF-α, and IL-2 production (**Figures 6A, B**), alongside a broad humoral response with statistically significantly elevated IgG1, IgG2a, IgG2b, and IgG2c levels compared to naïve group (**Figure 6C**). In contrast, metE formulated with AddaS03™ elicited a mixed immune response with a moderate Th1 CD4+ T cell response marked by TNF-α and IL-2 expression and a prominent Th2-skewed response, as evidenced by significantly increased IL-4 production compared to naïve group (**Figures 5A, B**). The antibody response for the metE-AddaS03™ group was consistent with this Th2 bias, showing statistically significant IgM and IgG1 responses but lower levels of IgG2a and IgG2c (**Figure 6C**). The metE DNA vaccine primarily induced a Th1-biased immune response of CD4+ T cells producing IFN-γ, TNF-α, and IL-2, but failed to elicit a strong antibody response, with no statistically significant increase in any of the measured immunoglobulin subclasses compared to naïve group (**Figures 6A–C**). We did not detect a statistically significant CD8^+^ T cell cytokine response following metE vaccination (**Supplementary Figure 4**). This may be attributed to the stimulation conditions used in the ICS assay, where the native metE protein was applied instead of a peptide pool, which failed to elicit a response. Both the AddaS03™ and AS01™ metE vaccine formulations induced significantly elevated IL-17 production compared to the naïve group, as detected by ICS and ELISpot assays (**Figures 6A, B**).

## 3 Discussion

Our study identified metE as a promising protective TB vaccine candidate through a combined immunopeptidomics and immunoinformatics approach. We demonstrated that metE, when formulated as a recombinant protein with either AS01™ or AddaS03™ adjuvants, provided significant protection against *MTB* in an *in vivo* challenge model, as evidenced by a reduction in bacterial burden compared to the naïve group. The metE-AS01™ formulation induced a robust Th1-skewed immune response, whereas the metE-AddaS03™ formulation elicited a mixed Th1/Th2 response. These findings suggest that metE could effectively enhance both cellular and humoral immune responses, both of which may contribute to protection against TB.

Building on our previous published work, we employed an immunopeptidomic approach to identify surface-presented mycobacterial antigens following infection of THP-macrophages with BCG or *MTB* (27). This approach led to the identification of 64 and 80 new mycobacterial antigens, respectively. We used an *in silico*-based approach to analyze the repertoire of MHC-bound peptides present in each identified antigen, allowing us to optimize candidate antigen selection based on epitope richness. As a result, three antigens (hisD, metE and mmpL12) were selected for protective efficacy evaluation. These antigens are present in both BCG and *MTB*, and their immunogenicity and protective efficacy has not been studied previously.

HisD (histidinol dehydrogenase) has been identified as a potential target for novel anti-TB drugs due to its essential role in histidine synthesis (41), countering immune cell-mediated histidine degradation to limit *MTB* proliferation (42). MetE (methionine synthase), found in cytosol, cell wall, cell membrane, and culture filtrate (43, 44), is essential for methionine synthesis, crucial for protein synthesis and cellular processes in *MTB* (45). Deleting the metE gene from *MTB* significantly hampers *MTB* growth (45, 46). Additionally, metE has been reported as a highly immunogenic antigen, playing a key role in the host immune response and being secreted at different stages of *MTB* growth (47). mmpL12 (mycobacterial membrane protein large) functions as a membrane transporter with broad substrate specificity, including glycolipid transportation (48). Although mmpL12 has been identified as a potential protective antigen through reverse vaccinology (49), which relies on computational tools to predict candidate antigens, its protective efficacy had not been tested prior to our study.

We focused first on evaluating the protective efficacy of these three antigens, and planned to perform detailed immunogenicity evaluation on any antigens found to be protective, given the lack of defined and validated immune correlates of protection for TB (50). Although antigen immunogenicity is important, it does not always translate to protection, as exemplified by numerous mycobacterial antigens that elicit IFNγ release but fail to confer protective effects against *MTB* challenge (51). Furthermore, CD4+ T cells can offer IFNγ-independent immunity against TB (52). The *ex vivo* MGIA provides an unbiased tool for high-throughput down-selection of promising protective antigens (53). The utility of the MGIA as a surrogate of protective efficacy has been demonstrated in mice, non-human primates and humans, allowing standardization and consistency in vaccine evaluation across different species (54–57). We demonstrated that mice immunized with metE, administered as a protein formulated with adjuvant, showed a significant protective effect compared to unvaccinated control mice.

Both metE-DNA and metE-AS01™ vaccine candidates induced a Th1-skewed phenotype, characterized by the expression of IFNγ, TNFα, and IL-2. In contrast, the metE-AddaS03™ vaccine candidate elicited a mixed Th1/Th2 immune response, marked by the production of TNFα, IL-2, and IL-4. The diverse CD4+ T cell phenotypes following metE immunization suggests multiple, potentially complementary, protective pathways, contingent on the delivery and adjuvant system. The consistent presence of TNFα and IL-2 indicates their significant roles in conferring protection independent of the presence of IFNγ (58).

The influence of the adjuvant system on the antibody response has also been observed. Studies in mice have shown that a high concentration of IgG2a is indicative of a Th1 response, while IgG1 presence suggests a Th2 response (59). This observation aligns with the Th1 and Th2 cellular responses triggered by metE-AS01™ and metE-AddaS03™ adjuvants, respectively. Similarly, in humans, the promising M72/AS01 subunit vaccine not only elicited effective T cell responses but also induced sustained production of IgG antibodies against the M72 antigen for up to three years (34, 60). This highlights the importance of choosing appropriate adjuvants in vaccine design to induce the desired immune response.

The challenges associated with interpreting murine spleen CFU data after aerosol challenge have previously been highlighted (61). It is unclear whether the mycobacteria are reaching the spleen through hematogenous disseminated infection or have disseminated into the lung capillaries and hence to the spleen. In our study, metE delivered as an adjuvanted protein conferred significant protection against pulmonary *MTB* infection, but this protection did not extend to the spleen. Several factors could potentially explain this difference. One explanation could be the susceptibility of the mice to *MTB* infection and/or the high dose of aerosol *MTB* used in our challenge, as evidenced by the CFU count of ~10^7^ CFU in the naïve group. Similar observations reported previously suggested that the inherent replicative ability of BCG might confer a more robust and sustained protective immunity against extrapulmonary TB in contrast to subunit vaccines (62–65). However, the encouraging results in the lung indicate that metE delivered systemically as adjuvanted protein can effectively induce protective immunity at the primary site of infection, the lung. This is important as some candidates have demonstrated lung-specific protection exclusively when administered via the pulmonary route compared to the parenteral administration (66, 67).

Our findings suggest that the magnitude of the T cell response is a significant factor in the level of protection conferred against *MTB* challenge. This is evident from the contrasting results of metE-DNA vaccination and metE-protein-adjuvant vaccination *in vivo*. The latter elicited a stronger T cell and antibody response, and metE-DNA vaccination, which induced a lower magnitude of response, provided no protection *in vivo* against *MTB* challenge. This observation aligns with clinical data comparing various candidate TB vaccines (MVA85A, AERAS-402, H1:IC31, H56:IC31, M72/AS01, ID93/GLA-SE, and BCG) (14), where all vaccines triggered antigen-specific CD4+ T-cell responses, predominantly featuring Th1 cytokine production. However, the magnitude of Th1 cells generated was a key differentiator among the vaccines evaluated. Despite similar cytokine profiles across all vaccines, M72/AS01 was distinguished by its ability to elicit a more robust memory CD4+ T-cell response. This suggests that the magnitude and durability of the CD4+ T cell response, rather than the specific cytokine profile, might be a more reliable indicator of protection against *MTB*.

## 4 Conclusions

Our immunopeptidomic approach has led to the identification of novel protective antigens for TB, and demonstrated the importance of delivery system, as well as antigen, in protection conferred by a subunit vaccine. One of the antigens identified, metE, conferred significant protection against *MTB* in mice when delivered as a protein/adjuvant vaccine candidate, suggesting its potential for inclusion in the next-generation of TB vaccines. Further exploration of heterologous prime-boost immunization regimes and evaluation in other preclinical animal models will confirm the role of this novel antigen in a candidate TB vaccine regimen. 

## 5 Methods

### 5.1 Cell culture

The THP-1 human leukemic monocytic cell line, obtained from the American Type Culture Collection, was cultured in RPMI-1640 medium (R0883-500ML, Sigma-Aldrich) supplemented with 10% heat-inactivated FCS, 2 mM L-glutamine, and 100 U/ml penicillin-streptomycin, and incubated at 37°C in a 5% CO_2_ atmosphere. BCG was grown in Middlebrook 7H9 broth (BD Biosciences, UK) with 50 μg/ml hygromycin (Merck Life Science UK Limited), 10% OADC enrichment (BD Biosciences, UK), 0.2% glycerol, and 0.05% tyloxapol (Sigma-Aldrich, UK), at 37°C with 200 rpm shaking. *MTB* was cultured under similar conditions but without shaking.

For infection experiments, 3 x 10^8^ THP-1 cells were differentiated into macrophages using 20 nM Phorbol 12-Myristate Acetate (PMA, Sigma-Aldrich, UK) overnight, then treated with 50 ng/ml IFNγ (Immuno Tools, Germany) and 1 μg/ml anti-IL10 (BD Biosciences, UK) for one day. *MTB* in exponential growth phase was centrifuged at 3,000 g for 10 minutes, washed, and resuspended in antibiotic-free medium. Bacteria were de-clumped by centrifugation at 300 g for 2 minutes and sonicated for 10 minutes in 10 cycles (30 seconds in a sonication bath and 30 seconds in ice). On the day of infection, THP-1 cells were washed and infected with mycobacteria at an MOI of five, then washed thrice after 4 hours to remove excess bacteria and resuspended in fresh antibiotic-free medium. After 24 hours, cells were harvested and stored at -20˚C.

To obtain bacteriological sterility prior to conducting immunopeptidomics experiments, the cell pellet was lysed using 2x lysis buffer and complete™ protease inhibitor cocktail (Roche) for 30 minutes on ice, with periodic shaking every 10 minutes. Post-lysis, the mixture was centrifuged at 500 g for 10 minutes to separate nuclei. The resulting supernatant was then filtered twice through centrifugal filters (0.22 µm, hydrophilic PVDF, 0.5 mL, Millipore) at 2000 g for 5 minutes each. The clear filtrate was collected in a new 15 ml Falcon tube for immunoprecipitation (IP).

### 5.2 IP of peptide-MHC complex

Our previous study consisted of THP-1 cells infected with live BCG, treated with heat-killed BCG, and stimulated with a cocktail of IFN-γ and anti-IL10, to improve MHC-II antigen expression (27). The cell lysates were subjected to two immunoprecipitation procedures to isolate HLA-DR, DQ, DP and HLA-A, B, C, using the L243 and W6/32 antibodies, respectively. In the present study, we subjected the same cell lysates to two additional immunoprecipitation procedures. Given that L243 is well known to strongly bind to HLA-DR, we immunoprecipitated the lysates with HLA-DP-specific antibody (B721), followed by the use of another pan-class II antibody (IVA12) to isolate HLA-DQ molecules (1 mg each per 1x10^8^ cells). Briefly, the antibodies were cross-linked to Protein A Sepharose beads with dimethyl pimelimidate (Sigma) and lysates were incubated overnight with each antibody. Beads were subsequently washed with 10 column volumes (cv) of 1x150 mM NaCl, 5 mM EDTA in 50 mM Tris, 1x150 mM NaCl in 50 mM Tris, 1x450 mM NaCl in 50 mM Tris and 50 mM Tris buffer without salt. Peptides bound to the HLA molecules were dissociated upon mild acid elution with 5 ml 10% acetic acid to denature α and β subunits of MHC molecules. The same protocol was applied to *MTB* IP with some modifications as follows. The IP order was B721 (HLA-DP), IVA12 (pan-MHC-II) and W632 (pan-MHC-I). A volume of 2 ml of Sepharose beads was used for 5 mg antibody per sample. Each sample was incubated with antibodies twice (first incubation overnight and second incubation for one hour).

HLA peptides were purified using reverse-phase high-performance liquid chromatography (RP-HPLC). Using the Ultimate 3000 HPLC system (Thermo Fisher Scientific), samples were injected into a 4.6x50 mm ProSwift RP-1S column (Thermo Fisher Scientific). They were then eluted over 10 minutes with a buffer gradient of 2% to 35% buffer B (0.1% TFA in acetonitrile) at a flow rate of 1000 µl/min. Fractions lacking ß2-microglobulin were pooled into two combined final fractions. These samples were dried, reconstituted in 20 µl of loading buffer (0.1% TFA, 1% acetonitrile), and stored at −20°C for subsequent mass spectrometry (MS) analysis.

For MS and data analysis, peptides were analyzed using nano-LC-MS-MS on an Ultimate 3000 RSLCnano System (PepMap C18 column, 2 µm, 75 µm × 50 cm; Thermo Fisher Scientific) connected to either an Orbitrap Fusion Lumos Tribrid or a Q Exactive HF-X mass spectrometer (both from Thermo Fisher Scientific). HLA-I peptides were separated with a 60-min gradient from 3% to 25% acetonitrile in 5% DMSO/0.1% formic acid, while HLA-II peptides used a gradient from 3% to 30% acetonitrile, both at 250nl/min flow rate.

For the Orbitrap Fusion Lumos Tribrid, full MS acquisition was at 120,000 resolution (300–1500 m/z range) with an AGC target of 400,000. Peptides were selected in a top-speed cycle of 2s, isolated at 1.2 amu, with an accumulation time of 120ms. MS-MS was at 30,000 resolution, AGC target of 300,000, using high-energy collisional dissociation (HCD) at 28V for peptides with 2–4 charge states and 32V for singly charged ions.

For the Q Exactive HF-X, full MS acquisition was at 120,000 resolution (320-1600m/z range) with an AGC target of 300,000. The top 20 precursor ions were fragmented at 1.6 amu isolation width for 120ms, resulting in a 2.5s cycle time. MS2 resolution was at 60,000 with an AGC target of 50,000, using HCD fragmentation at 25V collision energy.

For immunopeptidomics data analysis, MS data were processed using Peaks V8 and V10 (Bioinformatics Solutions). The spectra were matched against the proteomes of humans and BCG (Pasteur 1173P2) or *MTB* (H37Rv), both sourced from the UniProt database, to identify peptide spectrum matches (PSMs). Identified peptides and proteins were exported from Peaks with a minimum score threshold of -10logP=15. The exported data in Excel format were further analyzed to discern mycobacterial peptides and proteins, following these steps:

- Exclusion of all human peptides/proteins.
- Removal of any mycobacterial peptides/proteins found in the control sample from the infected samples.
- Detailed analysis of the mycobacterial peptides/proteins present in the infected samples.

### 5.3 T-cell MHC-II prediction

We adopted an in-silico approach utilizing immunopeptidomics and immunoinformatics tools to efficiently shortlist potential antigen candidates. The CD4+ T-cell epitopes were predicted using the NetMHCIIpan server (https://services.healthtech.dtu.dk/) with default settings for 15-mer peptides. The selected antigens were analyzed based on their entire sequences to determine which antigens contained a high number of T-cell epitopes. Binding affinity thresholds of 1% and 5% identified strong and weak binders, respectively. Predictions were made against 27 human HLA (DRB1*0101, DRB1*0301, DRB1*0401, DRB1*0405, DRB1*0701, DRB1*0802, DRB1*0901, DRB1*1101, DRB1*1201, DRB1*1302, DRB1*1501, DRB3*0101, DRB3*0202, DRB4*0101, DRB5*0101, DQA1*0501/DQB1*0201, DQA1*0501/DQB1*0301, DQA1*0301/DQB1*0302, DQA1*0401/DQB1*0402, DQA1*0101/DQB1*0501, DQA1*0102/DQB1*0602, DPA1*0201/DPB1*0101, DPA1*0103/DPB1*0201, DPA1*0103/DPB1*0401, DPA1*0301/DPB1*0402, DPA1*0201/DPB1*0501 and DPA1*0201/DPB1*1401). Given the relevance of mouse models at contributing to antigen presentation in the TB field, we included eight mouse MHC alleles (H-2-Dd, H-2-Db, H-2-Kd, H-2-Ld, H-2-Iad, H-2-IEd, H-2-Kb and H-2-IAb) relevant to the CB6F1 mouse strain. Mycobacterial proteins with a wide range of epitopes covering all these alleles were chosen for further analysis. HLA allele coverage was calculated by mapping the predicted epitopes against the aforementioned list of MHC alleles. Coverage for each antigen was determined based on the presence of predicted epitopes, categorized as either strong or weak binders, across the tested alleles. An antigen was considered to have 100% coverage if it contained epitopes for all tested alleles. For antigens with epitopes covering fewer alleles, coverage was calculated using the following equation:


$$Coverage\;\left(\%\right)=\;\left(\frac{Number\;of\;alleles\;with\;predicted\;epitopes}{Total\;number\;of\;tested\;alleles}\right)\;\times 100$$

Mycobacterial peptides derived from BCG and *MTB* were predicted against THP-1 cell HLA alleles, as reported by Bettencourt et al. (27), to predict the most likely associated allele, which is described as the “best hit.”

### 5.4 Non-homologous protein identification

To identify mycobacterial proteins that are non-homologous to the human proteome, each mycobacterial protein sequence was analyzed using the Vaxign server (https://violinet.org/vaxign/) for similarity with human proteins. The homology between sequences was assessed using the BLAST protein database, with smaller E-values indicating higher similarity.

### 5.5 *Escherichia coli* transformation

Competent *E. coli* bacteria were mixed with 5 μl DNA (~100ng, synthesized by Biomatik, USA) and incubated on ice for 20–30 minutes, heat-shocked at 42°C for ~45 seconds, and then cooled on ice for 2 minutes. Cultured in 500μl LB or SOC medium (New England Biolabs) at 37°C with shaking for 45 minutes, bacteria were then plated on LB agar with carbenicillin, using direct and streak-plate techniques. A selected colony was incubated in 10ml LB with carbenicillin at 37°C overnight with vigorous shaking. The starter culture was diluted 1:500 in fresh LB with carbenicillin and further incubated at 37°C overnight. Harvesting involved centrifuging at 4000g for 15 minutes and discarding the supernatant, with an option to freeze the pellet at -20°C.

### 5.6 Plasmid purification

Following the manufacturer’s instructions from the PureLink™ HiPure Expi Plasmid Gigaprep Kit (Thermo Fisher Scientific), bacterial pellets were processed with resuspension, lysis, and precipitation buffers. After centrifugation, the supernatant underwent filtration and vacuum application. Wash and endotoxin removal steps were followed by DNA binding and washing using a DNA-binding cartridge. The plasmids were then eluted with endotoxin-free buffer, precipitated with isopropanol, washed with ethanol, and dried. Finally, the DNA was resuspended in endotoxin/DNase-free water and its concentration measured using a NanoDrop™ UV-Vis Spectrophotometer (Thermo Fisher Scientific), with the purified DNA stored at -20°C.

### 5.7 Agarose gel electrophoresis

Agarose gel electrophoresis was performed to verify DNA insert sizes. Digested DNA in 0.5ml tubes was incubated at enzyme-specific temperatures for 5–15 minutes. A 1% agarose gel with 10μl of SYBR Safe stain was set in a comb-equipped tray and solidified for 15–20 minutes. The gel, placed in a tank with 1x TAE buffer, was loaded with the DNA mixture (25μl sample + 5μl 6x loading dye) and 5μl ladder, then run at 100V for 50 minutes. Post-electrophoresis, the gel was imaged using the Gel Doc EZ System (Bio-Rad).

### 5.8 Flow cytometry

To verify intracellular expression of antigens by the pDNA3.1 plasmid human embryonic kidney cells (293T) were cultured in six-well plates in RPMI-1640 medium (R0883-500ML, Sigma-Aldrich) supplemented with 10% heat-inactivated FCS, 2mM L-glutamine, and 100 U/ml penicillin-streptomycin at 37°C until they reached 70-80% confluency. Then, the cells were transfected with 1 µg of pDNA3.1 plasmid using a 1:3 DNA: PEI ratio for 2–3 hours. Post-transfection, cells were incubated for 24 hours in complete RPMI medium. Cells were harvested with 0.5 ml trypsin, fixed in 100 μl of 4% paraformaldehyde, and permeabilized with 100 μl of 0.1% saponin. They were stained with PE-anti-his-tag antibody (1:50 dilution) and washed. The analysis was conducted using an LSRII™ flow cytometer (BD Biosciences), with data processed in FlowJo^®^ software.

### 5.9 Animal work

All procedures were performed in accordance with the UK Animals (Scientific Procedures) Act 1986, under project license number 30/2889 and P9804B4F1, granted by the UK Home Office. All procedures followed institutional and national ethical guidelines, approved by the University of Oxford’s Animal Welfare and Ethical Review Board (AWERB), and adhered to the Animal Research: Reporting of *In Vivo* Experiments (ARRIVE) guidelines.

#### 5.9.1 Immunization experiments

Specific-pathogen-free female CB6F1 mice (6–9 weeks old, Charles River UK Ltd.) were used for all experiments. All injections were performed under short-term anesthesia using vaporized isoflurane. Mice were humanely euthanized by cervical dislocation at the end of each experiment. BCG (Pasteur strain) used for vaccination was prepared in-house in 7H9 broth supplemented with 10% ADC enrichment medium and 0.05% Tween 80 (Becton Dickinson, UK). All DNA vaccines were synthesized by Biomatik (USA), and MetE protein was synthesized by BiologicsCorp (USA). AddaS03™ (InvivoGen, UK) and AS01 (obtained from SHINGRIX vaccine, Oxford University Hospitals) were used as adjuvants.

In the first experiment MGIA, mice were divided into six groups of eight. One group received 50 µL intradermal BCG (3.5 × 10^5^ CFU/mouse), administered once to the ears (25 µL per ear). Four groups received DNA vaccines (hisD, MetE, mmpL12; 100 µg/100 µL phosphate-buffered saline [PBS]/mouse), delivered intramuscularly into each thigh (50 µL per thigh) three times at two-week intervals. One group served as an unvaccinated control. Spleens and blood were collected two weeks after the final vaccination.

In the second MGIA experiment, mice were divided into five groups of eight. One group received 50 µL intradermal BCG (3.5 × 10^5^ CFU/mouse), administered once to the ears (25 µL per ear). Three groups received intramuscular MetE vaccinations: metE protein alone (1 µg/100 µL PBS/mouse), metE-DNA (100 µg/100 µL PBS/mouse), and metE protein combined with AddaS03™ adjuvant at a 1:1 volume ratio (1 µg/100 µL PBS/mouse). Vaccinations were administered into each thigh (50 µL per thigh) three times at three-week intervals. One group served as an unvaccinated control. Spleens and blood were collected three weeks after the final vaccination.

In the Immunogenicity experiment, mice were divided into four groups of five. Three groups were vaccinated three times at three-week intervals with the following formulations: metE-DNA (100 µg/100 µL PBS/mouse), MetE protein alone (1 µg/100 µL PBS/mouse), and 1 µg metE protein combined with AddaS03™ adjuvant at a 1:1 volume ratio in a total of 100 µL PBS/mouse, and AS01 adjuvant (5 µg monophosphoryl lipid A [MPL]/5 µg QS-21 in a total of 100 µL PBS/mouse, representing 1/10th of the human dose of AS01). Vaccinations were delivered into each thigh (50 µL per thigh). One group served as an unvaccinated control. Spleens and blood were collected three weeks after the final vaccination.

In the MTB-Challenge experiment, mice were divided into six groups of eight. One group received 50 µL intradermal BCG (Pasteur strain, 3.5 × 10^5^ CFU/mouse), administered to the ears (25 µL per ear). Five additional groups were vaccinated three times at three-week intervals with the following formulations: AddaS03™ adjuvant at a 1:1 volume ratio in a total of 100 µL PBS/mouse, AS01 adjuvant (5 µg MPL/5 µg QS-21 in a total of 100 µL PBS/mouse), metE-DNA (100 µg/100 µL PBS/mouse), 1 µg metE protein combined with AddaS03™ adjuvant at a 1:1 volume ratio in a total of 100 µL PBS/mouse, and AS01 adjuvant (5 µg MPL/5 µg QS-21 in a total of 100 µL PBS/mouse). Vaccinations were delivered into each thigh (50 µL per thigh). One group served as an unvaccinated control. Four weeks after the final vaccination, all mice were infected with 1 × 10^6^ CFU/ml of MTB Erdman K01 (TMC107, BEI Resources, USA) using a Biaera Aero-MP-controlled nebulizer (Biaera Technologies, USA). The bacterial load in the lungs was determined to be 50–100 CFU one hour post-infection in two mice. At the end of the experiment, four weeks post-infection, lungs and spleens were collected and homogenized in reinforced tubes (Stretton Scientific) using a Precellys 24 homogenizer. Homogenates were serially diluted in PBS, plated on Modified 7H11 plates (Animal and Plant Health Agency, UK), incubated at 37°C for 4–6 weeks, and then enumerated for CFUs.

#### 5.9.2 MGIA

Splenocytes were isolated by mashing spleens in a six-well plate, rinsing with PBS, and filtering through a 70 μm strainer into a 50 ml Falcon tube. Post-centrifugation at 350 g for 5 minutes, cells were lysed in 3 ml ACK buffer for 5 minutes, then diluted to 35 ml with RPMI with HEPES buffer and without antibiotics (RPMI-MGIA) and centrifuged again. The pellet was resuspended in 2 ml RPMI-MGIA, adjusting splenocyte concentration to 3x10^6^ or 5x10^6^ in 600μl for co-culture with BCG (300 or 500 CFU) in 48-well plates, and incubated for 96 hours at 37°C with CO_2_.

Well contents were transferred to 2 ml tubes and centrifuged at 12,000 rpm for 10 minutes. Concurrently, 500 µl sterile water was used to lyse remaining cells in each well. Supernatants were discarded, and water lysates were combined with the tube contents. After pulse-vortexing, the mixture was transferred to BACTEC mycobacteria growth indicator tubes (MGITs) with PANTA (polymyxin-B, amphotericin-B, nalidixic acid, trimethoprim, azilocillin) antibiotics and Oleic Albumin Dextrose Catalase (OADC) enrichment broth (Becton Dickinson, UK) until positivity was reached and expressed as TTD.

#### 5.9.3 Enzyme-linked immunosorbent assay

Blood samples from mice were collected into 2 ml tubes, stored overnight at 4°C, and centrifuged at 2000 g for 10 minutes. Serum was extracted and stored at -20°C. Maxisorp 96-well plates (Thermo Fisher Scientific) were coated with 2.5 µg/ml recombinant metE in PBS and incubated overnight at room temperature. After washing with PBS/Tween-20 and blocking with casein buffer for 1–2 hours, diluted mouse sera (initial dilution 1:100) were serially diluted and incubated for 2 hours. Plates were washed and treated with alkaline phosphatase-conjugated anti-mouse secondary antibodies (BioRad) at a 1:3000 ratio for 1 hour. Development buffer (1mg/ml 4-nitrophenylphosphate in diethanolamine buffer) was added, and optical density was measured at 405nm using a BioTeK Microplate Reader. Adjusted optical densities were obtained by subtracting blank well averages from sample averages.

#### 5.9.4 Enzyme-linked immunospot

Splenocytes were used in ELISpot assays to measure cytokine secretion upon metE stimulation. MultiScreen-IP filter plates (Millipore) were coated with 50 μl of 10 μg/ml anti-mouse cytokine antibodies (IFNγ, IL-2, IL-4, IL-17; Mabtech) and stored at 4°C overnight. After washing and blocking with complete media for 2 hours at 37°C, 50 μl of splenocyte suspensions (2.5 x 10^5^ cells) and 50 µl of either media (negative control) or 10 μg/ml metE (dissolved in the same media) were added to each well. Following overnight incubation, plates were washed and treated with 50 µl/well of 1:1000 diluted biotinylated goat anti-mouse IgG (Mabtech) for 2 hours. After subsequent washing, 50μl/well of 1:1000 diluted streptavidin-ALP (Mabtech) was added, incubated for 1 hour, and then developed with 50 μl/well of AP Conjugate sub kit (BioRad) for 5–10 minutes. Post-wash, spots were counted using an ELISpot reader (AID Germany), with results reported as spot forming units (SFU) per 1x10^6^ cells, adjusting for unstimulated cell counts.

#### 5.9.5 Intracellular cytokine staining

Splenocytes were prepared and stimulated in 96-well U-bottom plates with 1 million cells/well. Stimulation involved 10 μg/ml metE protein for metE vaccinated groups, 10 μg/ml tuberculin-purified protein derivative (PPD) for the BCG vaccinated group, and complete RPMI-1640 medium for unstimulated controls. This was accompanied by anti-CD28 and anti-CD49d (1 μg/ml each, BioLegend Inc.). Plates were incubated for 2 hours at 37°C with 5% CO2, followed by 4–6 hours with 1 μl/ml GolgiPlug (BD Biosciences), and then overnight at 4°C.

Post-incubation, cells were centrifuged, washed with PBS, and stained. Unstained wells received 10μl PBS, while others received 10 μl live/dead stain (1:200, Invitrogen, UK) for 10 minutes. Surface staining was conducted with 40 μl of antibody-cocktail 1 (**Table 2**), followed by a 30-minute incubation at 4°C. After washing with BSA/PBS, cells were fixed with Cytofix/Cytoperm (BD Biosciences) for 10 minutes and permeabilized with Perm/Wash solution.

**Table 2:** Composition and dilution of antibodies used for surface and intracellular staining.

Staining	Antibody cocktail	Dilution
Surface- cocktail 1	FC blocker	1:50
	PE-CF594 rat anti-mouse CD45R (eBioscience)	1:300
	TCR beta antibody, eFluor TM 450 (H57-597), (eBioscience)	1:100
	Brilliant Violet 650(TM) anti-mouse CD4 (BioLegend Inc)	1:100
	CD8a antibody, APC-eFluor TM 780 (53-6.7), (eBioscience)	1:300
Intracellular- cocktail 2	IFNγ antibody, APC (XMG1.2), (eBioscience)	1:200
	TNFα antibody, Alexa Fluor 488 (MP6-XT22), (eBioscience)	1:200
	IL-2 antibody, PE-cyanine7 (JES6-5H4), (eBioscience)	1:200
	IL-17A antibody, PE (eBio17B7), (eBioscience)	1:200

Intracellular Cytokine Staining (ICS):

Post-permeabilization, cells underwent intracellular cytokine staining using 40 μl of antibody-cocktail 2 (**Table 2**) in Perm/Wash solution, incubated for 30 minutes at 4°C. Following two washes with Perm/Wash and PBS/BSA solution, cells were resuspended in 80 μl PBS/BSA and analyzed on an LSR-II™ flow cytometer with FACSDiva™ software (BD Biosciences). Data were processed using FlowJo^®^ software, applying the gating strategy outlined in **Supplementary Figure 5**.

### 5.10 Statistical analyses

Statistical analysis was conducted using GraphPad Prism version 8. The Mann-Whitney test compared two groups, while the Kruskal-Wallis test with Dunn’s multiple comparison post-test was utilized for analyses involving multiple groups. A p-value of less than 0.05 was considered statistically significant.

## Data Availability

The raw data supporting the conclusions of this article will be made available by the authors, without undue reservation.
