# Supplementary material for: MetE: a promising protective antigen for tuberculosis vaccine development
Source: Front Immunol. 2025 Jul 21;16:1593263. doi: 10.3389/fimmu.2025.1593263 (PMC12319054; doi:10.3389/fimmu.2025.1593263)
Supplement: Supplementary file 1 [file DataSheet1.docx]

**MetE: A Promising Protective Antigen for Tuberculosis Vaccine Development**

Salem Salman Almujri¹^,^², Elena Stylianou¹, Annalisa Nicastri¹^,^³, Iman Satti¹, Marcellus Korompis¹, Shuailin Li¹, Christopher J. De Voss¹, Marco Polo Peralta Alvare¹, Rachel Tanner¹^,^⁴, Paulo J. G. Bettencourt¹^,^⁵, Nicola Ternette¹^,^⁶, Helen McShane¹*

¹ The Jenner Institute, University of Oxford, Oxford, United Kingdom
² Department of Pharmacology, College of Pharmacy, King Khalid University, Asir-Abha 61421, Saudi Arabia
³ Chester Beatty Laboratories, The Institute of Cancer Research, London, UK
⁴ Department of Biology, University of Oxford, Oxford, United Kingdom
⁵ Universidade Católica Portuguesa, Faculty of Medicine, Center for Interdisciplinary Research in Health, Lisbon 1649-023, Portugal
⁶ School of Life Sciences, University of Dundee, Dundee, Scotland, UK


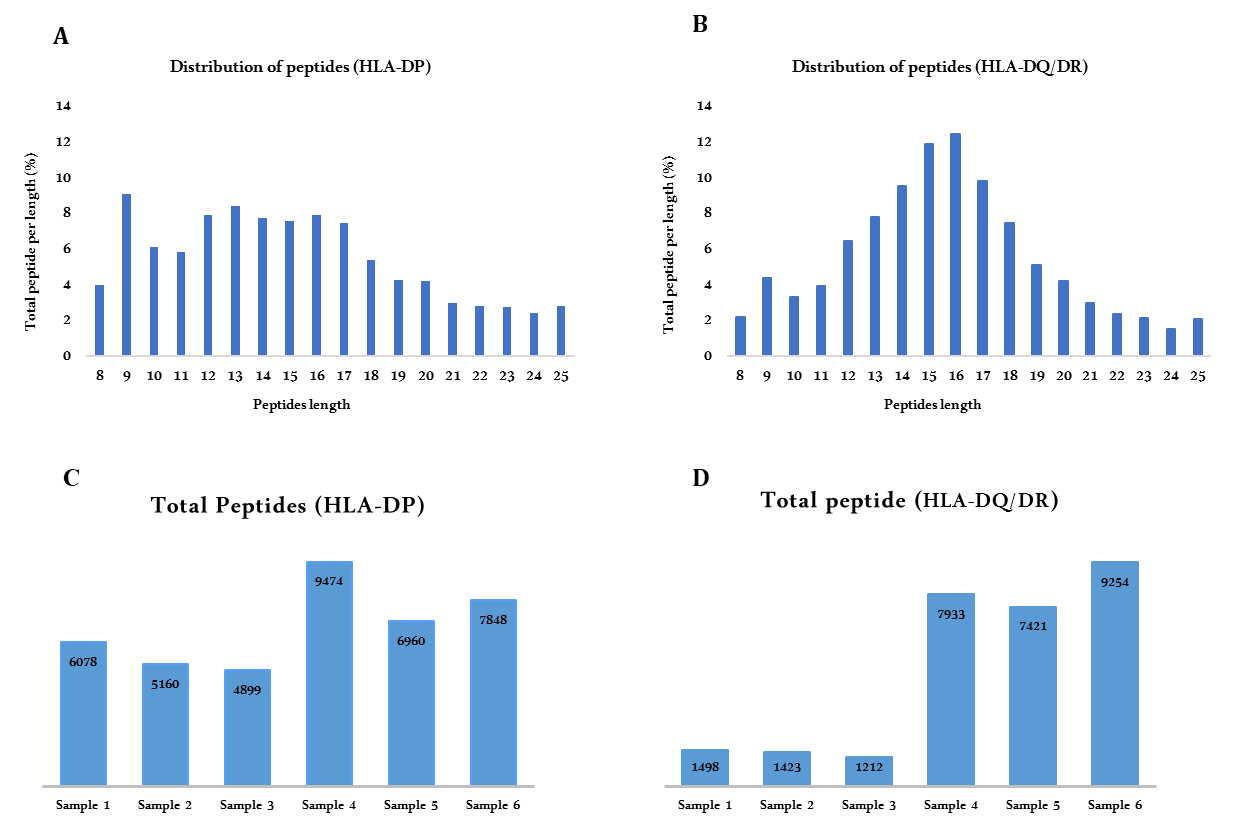


**Supplementary Figure 1: Immunopeptidome Characterization of Peptide Length and Distribution of BCG Experiments.** Panels A and B show the percentage of peptide length distribution obtained from B721 (HLA-DP specific) and IVA12 (pan-HLA-II) antibodies. Panels C and D display the total number of peptides identified with B721 and IVA12 antibodies across samples.


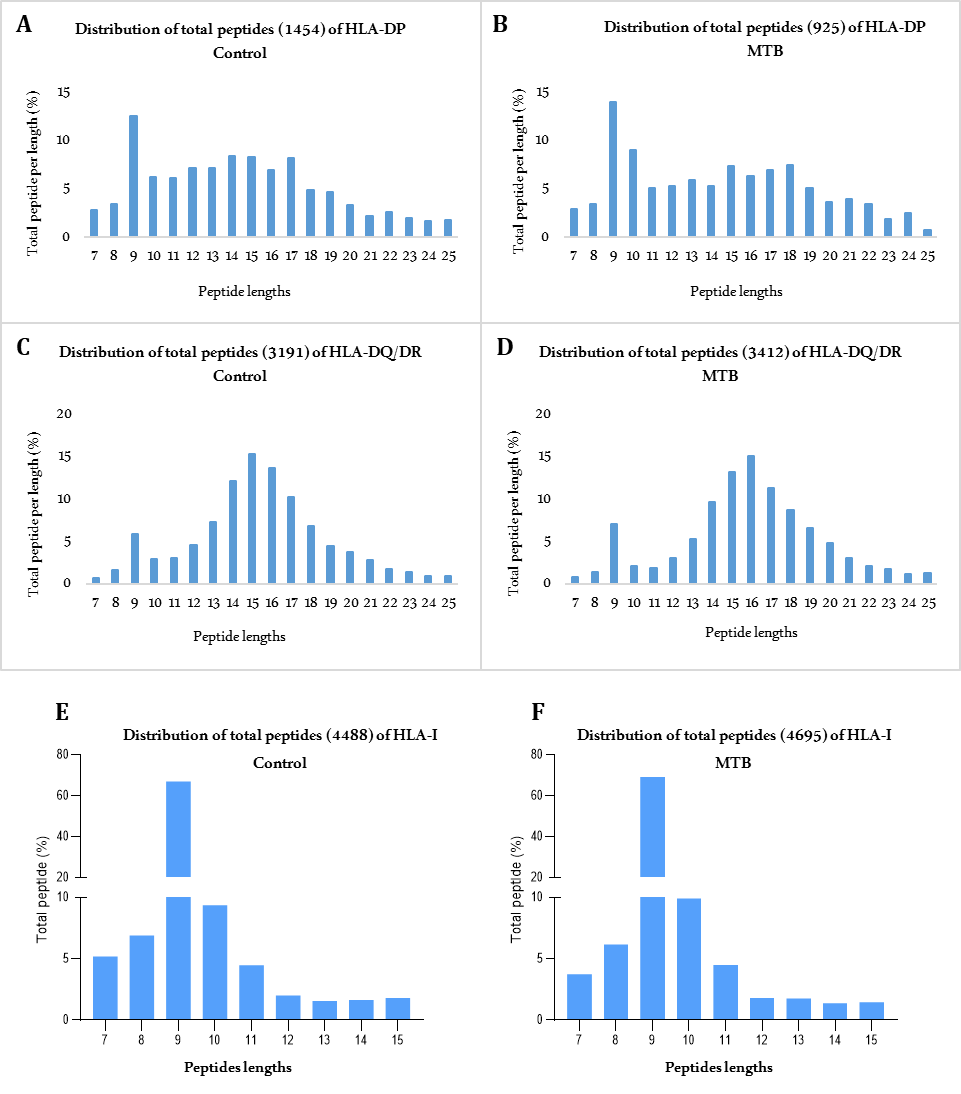


**Supplementary Figure 2: Immunopeptidome Characterization of Peptide Length and Distribution of MTB Experiments.** Panels A, C, and E show the percentage distribution of peptide lengths obtained from control samples using B721 (HLA-DP specific), IVA12 (pan-HLA-II), and W6/32 (pan-HLA-I) antibodies. Panels B, D, and F show the percentage distribution of peptide lengths obtained from MTB-infected samples using the same antibodies. The numbers in parentheses represent the total number of identified peptides.


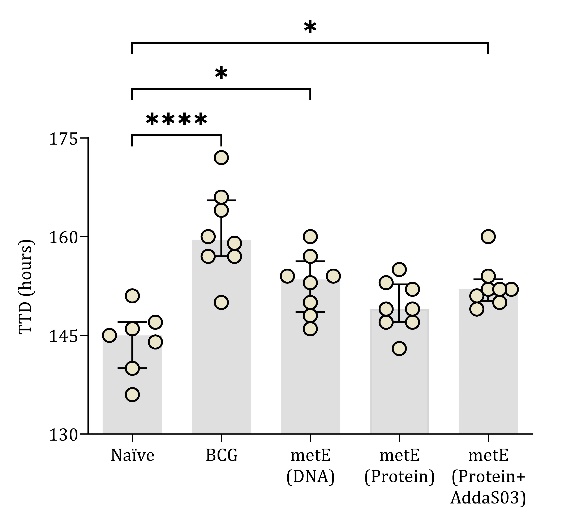


5x10^6^ splenocytes, 300 CFU BCG

**Supplementary Figure 3: TTD values of mycobacterial growth as measured by MGIA**. In the second experimental setup, TTD values were derived from 5x10^6^ mouse splenocytes co-cultured with BCG, inoculated 300 CFU. Splenocytes originated from naïve mice and those vaccinated with BCG, metE alone, metE-AddaS03™ , or metE-DNA. Plotted circles represent individual mice, lines representing median data, and whiskers indicating interquartile ranges (IQR). For identifying significant deviations compared to the naïve control, a combination of the Kruskal-Wallis and Dunn’s multiple comparisons tests was applied. Significance levels are denoted as * for P values <0.05, and **** indicating P values <0.0001.

B

A


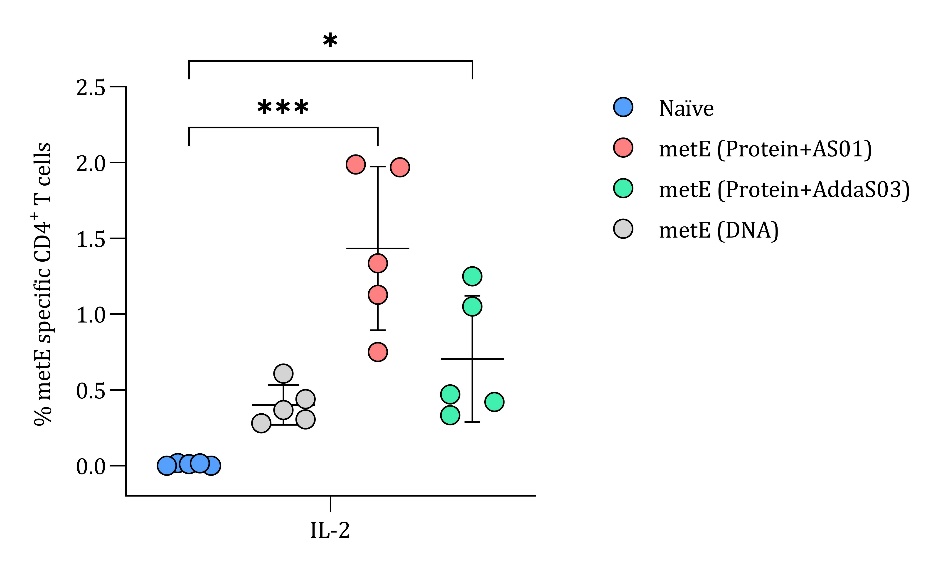

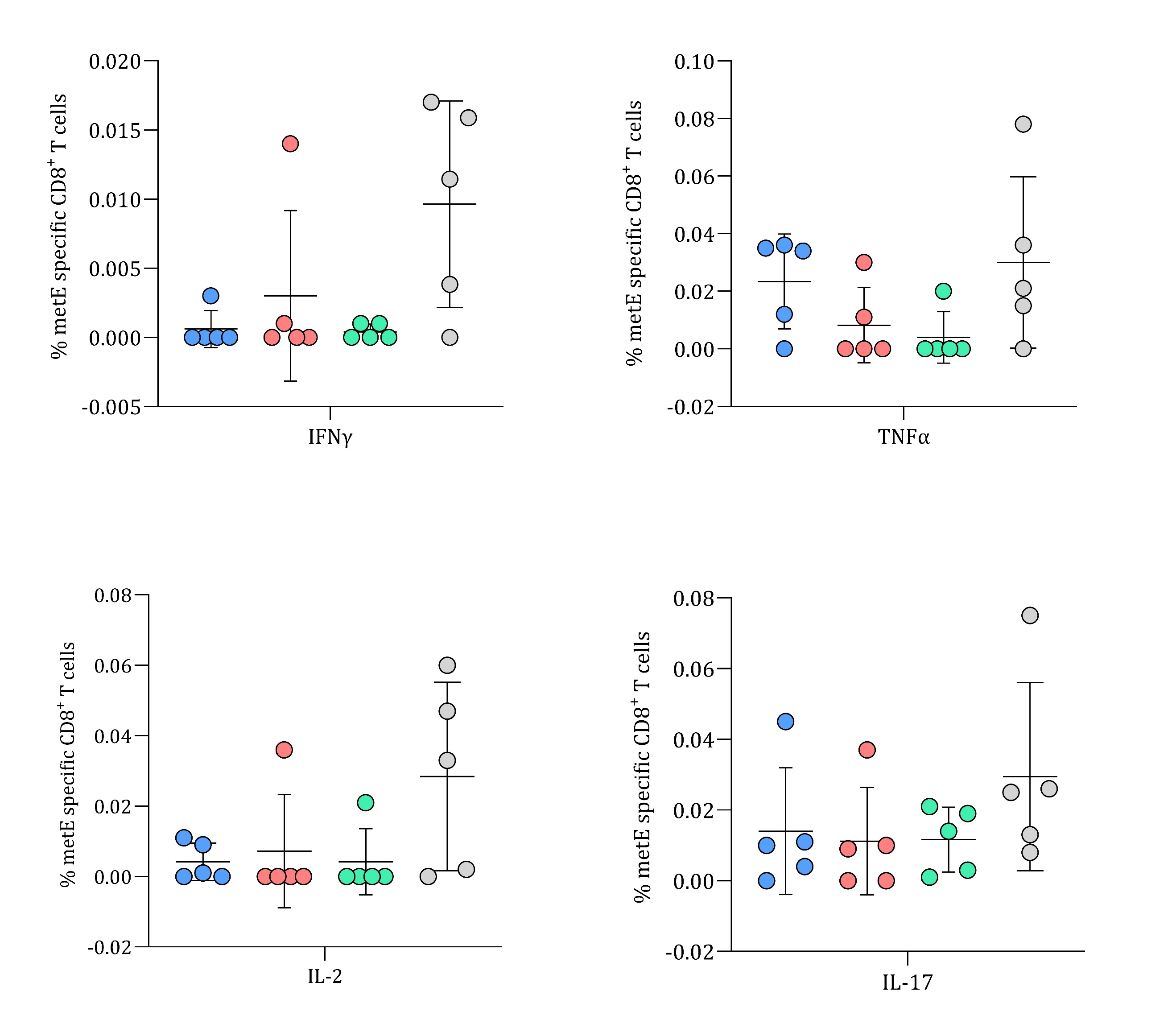

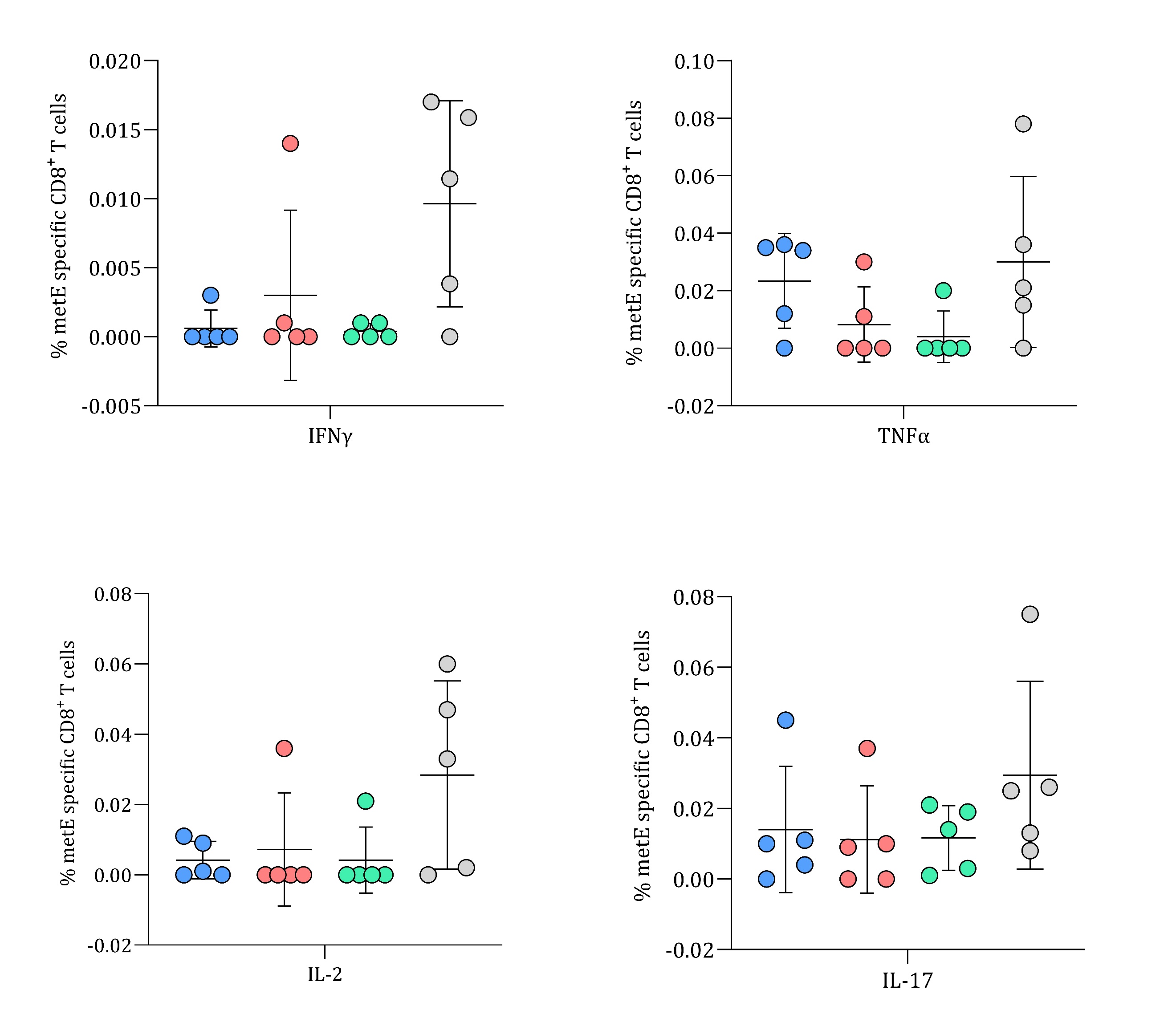


D

C

**Supplementary Figure 4. CD8⁺ T cell cytokine responses following MetE stimulation.**
Mouse splenocytes were stimulated overnight with metE protein and subsequently stained for CD8⁺ surface markers and intracellular cytokines. Cytokine expression was analyzed by flow cytometry. Panels show the perecentage of CD8⁺ T cells producing: (A) IFN-γ, (B) TNF-α, (C) IL-2, and (D) IL-17. Each plotted circle represents individual mice, with lines denoting median values. Significant differences compared to the naïve control were identified using the Kruskal-Wallis test with Dunn’s multiple comparisons test.


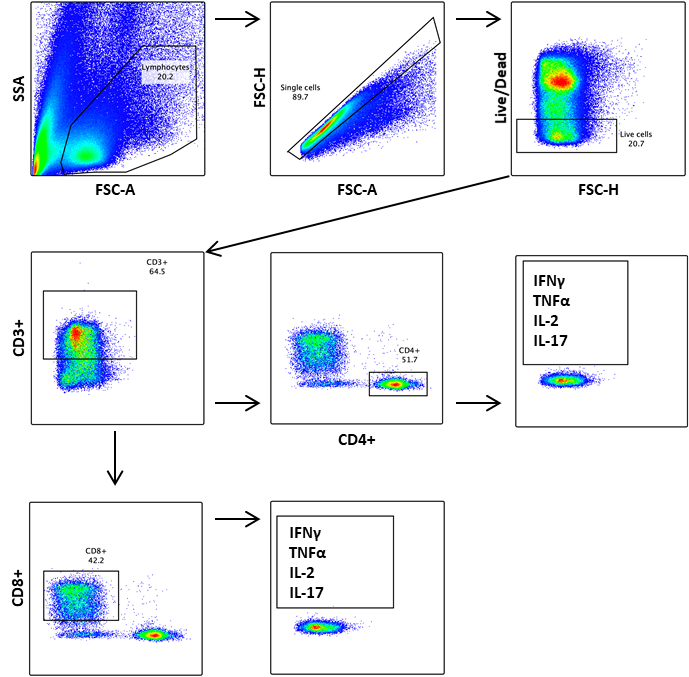


**CD8+**

**Cytokine of interest**

**CD4+**

**CD8+**

**Cytokine of interest**

**CD4+**

***Supplementary Figure 5: cytometric gating strategy for ICS.*** *Following the identification of lymphocytes through forward scatter (FSC) and side scatter (SSC), single cells were selected, and from these, viable cells were distinguished. Subsequently, the population expressing CD3+ was isolated, leading to the identification of both CD4+ and CD8+ subsets. The expressions of cytokines (IFNγ, TNFα, IL-2, and IL-17) were then measured independently within the CD4+ and CD8+ cell groups.*

**Supplementary Table 1:** Identified BCG peptides and corresponding proteins.

| **Number** | **Peptide** | **Peaks score**  **(-10lgP)** | **Length** | **Gene** | **Protein** | **Accession** | **Predicted THP-1 cell HLA allele (best hit)** | **Predicted affinity (IC50)** |
| --- | --- | --- | --- | --- | --- | --- | --- | --- |
| IP: B721  Sample 2: HK-BCG | | | | | | | | |
| 1 | WDNGTTHD | 32.83 | 8 | ctpI | Probable cation-transporter atpase I | A0A0H3M0P5 | HLA-B*35:01 | 39648.2 |
| 2 | TVRCVSGTVEL | 23 | 11 | BCG_2418 | Uncharacterized protein | A0A0H3MCK0 | HLA-C*03:03 | 21408.2 |
| 3 | KHRGLSFL | 21.91 | 8 | fadE17 | Probable acyl-CoA dehydrogenase | A0A0H3M7B4 | HLA-A*24:02 | 29214.9 |
| 4 | KDVSGHVITL | 20.04 | 10 | hisD | Histidinol dehydrogenase | A0A0H3M564 | HLA-A*02:01 | 2567.6 |
| 5 | KVVVVGGGT | 18.63 | 9 | cysG | Possible multifunctional enzyme siroheme synthase cysG | A0A0H3M9R7 | HLA-A*24:02 | 24537.7 |
| 6 | KAAPAKKAAPAK | 18.16 | 12 | hbhA | Heparin-binding hemagglutinin | A1KFU9 | DQA1*01:02/DQB1*06:02 | 13113.15 |
| 7 | TGSMHDLLMSNFFA | 16.95 | 14 | pgi | Glucose-6-phosphate isomerase | A1KH80 | DPA1*01:03/DPB1*0201 | 1129.06 |
| 8 | KKAPAKASET | 16.93 | 10 | rplV | 50S ribosomal protein L22 | A1KGI7 | HLA-A*24:02 | 41183.4 |
| 9 | TTYTLEYDG | 15.96 | 9 | PE1 | PE family protein | A0A0H3M9W4 | HLA-B*35:01 | 18697.8 |
| 10 | VLSLLGMVLLMLGTGRL | 15.24 | 17 | BCG_3867c | Probable conserved transmembrane protein | A0A0H3MFQ9 | DRB1*01:01 | 87.83 |
| IP: B721  Sample 3: live BCG | | | | | | | | |
| 1 | KHRGLSFL | 25.07 | 8 | fadE17 | Probable acyl-CoA dehydrogenase | A0A0H3M7B4 | HLA-A*24:02 | 29214.9 |
| 2 | TVAIQNKAI | 19.57 | 9 | BCG_3870c | Galactofuranosyl transferase | A0A0H3MAQ2 | HLA-B*35:01 | 14713.6 |
| 3 | ELATSRRPDHLHGLATQL | 18.74 | 18 | BCG_1984 | Uncharacterized protein | A0A0H3MEB3\|A0A0H3M474 | DQA1*01:02/DQB1*06:02 | 8185.82 |
| 4 | CAAVCTGCVAVVLAPVSLA | 17.67 | 19 | BCG_0349 | Possible conserved exported protein | A0A0H3M181 | DQA1*01:02/DQB1*06:02 | 237.34 |
| 5 | KATMRISA | 17.58 | 8 | treZa | Maltooligosyltrehalose trehalohydrolase TreZ | A0A0H3MAJ3 | HLA-C*03:03 | 36911.3 |
| 6 | TVRCVSGTVEL | 17.4 | 11 | BCG_2418 | Uncharacterized protein | A0A0H3MCK0 | HLA-C*03:03 | 21408.2 |
| 7 | VAGPVPVAGHLT | 15.62 | 12 | BCG_0616c | Uncharacterized protein | A0A0H3MAR4 | DQA1*01:02/DQB1*06:02 | 5432.85 |
| 8 | LRHRIRFGA | 15.53 | 9 | BCG_0944 | Probable monooxygenase | A0A0H3M920 | HLA-A*02:01 | 30044.6 |
| 9 | LLARRPVVH | 15.45 | 9 | BCG_3268c | Probable conserved transmembrane transport protein | A0A0H3MAR2\|A0A0H3MBW3 | HLA-B*35:01 | 14918.6 |
| 10 | DFDGAADAGFTAPATTL | 15.04 | 17 | PPE55a | PPE Family protein [first part] | A0A0H3M8U0 | DQA1*01:02/DQB1*06:02 | 2106.3 |
| 11 | KHRGLSFL | 25.07 | 8 | fadE17 | Probable acyl-CoA dehydrogenase | A0A0H3M7B4 | HLA-A*24:02 | 29214.9 |
| IP: B721  IP: B721  Sample 5: + IFNγ / anti-IL-10 + HK-BCG | | | | | | | | |
| 1 | ADIAIQAEQFAVIKK | 45.19 | 15 | clpP1 | ATP-dependent Clp protease proteolytic subunit | A0A0H3M791 | DPA1*01:03/DPB1*02:01 | 10.6 |
| 2 | EVDEDTSAYD | 21.33 | 10 | cysA2 | Sulfurtransferase | A0A0H3MB67\|A0A0H3M8T4 | HLA-B*35:01 | 1075.1 |
| 3 | EVNSARIYAGA | 19.71 | 11 | PPE13 | PPE family protein | A0A0H3M4M6 | HLA-A*02:01 | 35422.1 |
| 4 | TLRLPLDV | 18.38 | 8 | BCG_1012 | Possible magnesium chelatase | A0A0H3M4V4 | HLA-A*02:01 | 7092.9 |
| 5 | IKYNGEEYLIL | 17.65 | 11 | groS | 10 kDa chaperonin | A1KPA9 | HLA-A*24:02 | 1629.9 |
| 6 | FVRSRPELKLPDLELIFAP | 16.86 | 19 | BCG_1338 | Probable dehydrogenase FAD flavoprotein gmc oxidoreductase | A0A0H3M4G4 | DPA1*01:03/DPB1*02:01 | 749.38 |
| 7 | ELATSRRPDHLHGLATQL | 16.24 | 18 | BCG_1984 | Uncharacterized protein | A0A0H3MEB3\|A0A0H3M474 | DQA1*01:02/DQB1*06:02 | 8185.82 |
| 8 | LATLFGDGVLRPLPV | 16.09 | 15 | pks12 | Probable polyketide synthase pks12 | A0A0H3M7L7 | DPA1*01:03/DPB1*02:01 | 168.62 |
| 9 | TGFYNTGDVNT | 16.08 | 11 | PPE8 | PPE family protein | A0A0H3M207 | HLA-A*02:01 | 39408.3 |
| 10 | KPADGVPPPPLNTKLPEDPPP | 15.87 | 21 | BCG_2181c | Probable conserved proline rich membrane protein | A0A0H3MBV1 | DRB1*01:01 | 19469.55 |
| 11 | KFARITLGT | 15.67 | 9 | nuoG | NADH-quinone oxidoreductase | A0A0H3MAH2 | HLA-A*24:02 | 14573.7 |
| 12 | SAVTAGLCRALAR | 15.36 | 13 | cobQ | Cobyric acid synthase | A1KF76 | DQA1*01:02/DQB1*06:02 | 593.19 |
| IP: B721  Sample 6: + IFNγ / anti-IL-10 + live BCG | | | | | | | | |
| 1 | ADIAIQAEQFAVIKK | 31.37 | 15 | clpP1 | ATP-dependent Clp protease proteolytic subunit | A0A0H3M791\|A0A0H3M791_MYCBP | DPA1*01:03/DPB1*02:01 | 10.6 |
| 2 | KAAPAKKAAPAK | 22.72 | 12 | hbhA | Heparin-binding hemagglutinin | A1KFU9 | DQA1*01:02/DQB1*06:02 | 13113.15 |
| 3 | APTNHAGV | 19.78 | 8 | BCG_2590 | Conserved hypothetical alanine and leucine rich protein | A0A0H3MCX2 | HLA-B*35:01 | 30626.5 |
| 4 | AVDPTVRVQ | 19.56 | 9 | BCG_0467c | Uncharacterized protein | A0A0H3MAG6 | HLA-B*35:01 | 27632.5 |
| 5 | AILRLPLT | 18.71 | 8 | BCG_2874 | Uncharacterized protein | A0A0H3MGJ6 | HLA-A*02:01 | 23491.8 |
| 6 | KPAKAAKPAL | 18.22 | 10 | BCG_3557c | Conserved hypothetical mce associated alanine and valine rich protein | A0A0H3M9N5 | HLA-B*35:01 | 5232.2 |
| 7 | LLIDRLSVQ | 17.05 | 9 | BCG_2206c | Uncharacterized protein | A0A0H3M6M1 | HLA-A*02:01 | 8975.4 |
| 8 | TALLQLLSA | 16.52 | 9 | BCG_1349c | Uncharacterized protein | A0A0H3M4H3 | HLA-A*02:01 | 5769.5 |
| 9 | GFYNTGSYNTGGFN | 16.03 | 14 | PPE8 | PPE family protein | A0A0H3M207 | DPA1*01:03/DPB1*02:01 | 10499.35 |
| 10 | FGTFQLTMFGVGATI | 15.53 | 15 | rocE | Probable cationic amino acid transport integral membrane protein rocE | A0A0H3M616 | DPA1*01:03/DPB1*02:01 | 45.51 |
| 11 | KAAQVATTLT | 15.05 | 10 | BCG_3311 | Nucleoside triphosphate pyrophosphatase | A1KNT4 | HLA-C*03:03 | 8125.6 |
| IP: IVA12  Sample 2: HK-BCG | | | | | | | | |
| 1 | WDNGTTHD | 24.84 | 8 | ctpI2 | Probable cation-transporter atpase I ctpI | A0A0H3M0P5 | HLA-B*35:01 | 39648.2 |
| 2 | KKAAPAKKAAPAK | 23.04 | 13 | hbhA | Heparin-binding hemagglutinin | A1KFU9 | DRB5*01:01 | 9245.4 |
| 3 | TFSGLLDRFGRGKGCDICKPVVAS | 17.42 | 24 | nirB | Probable nitrite reductase | A0A0H3M1Q9 | DRB1*01:01 | 1136.57 |
| 4 | PGDMPGGTAAADAAHTED | 16.92 | 18 | grpE | Protein GrpE | A1KFH3 | DQA1*01:02/DQB1*06:02 | 1378.28 |
| 5 | TADQRVLAIRLTNGSSLLISKSLKP | 16.35 | 25 | mprB | Signal transduction histidine-protein kinase/phosphatase MprB | A1KHB8 | DRB1*01:01 | 73.2 |
| IP: IVA12  Sample 3: live BCG | | | | | | | | |
| 1 | KPLVRHTVHT | 19.84 | 10 | nrp | Probable peptide synthetase nrp | A0A0H3M1C1 | HLA-A*02:01 | 11320.5 |
| 2 | KAPAKKAATKAPAKK | 18.28 | 15 | hupB | Probable DNA-binding protein HU homolog hupB | A0A0H3M7X4 | DRB5*01:01 | 486.47 |
| 3 | KALMSIGKVITASAAT | 16.7 | 16 | mmpL12 | Probable conserved transmembrane transport protein mmpL12 | A0A0H3M689 | DQA1*01:02/DQB1*06:02 | 62.5 |
| 4 | KATLMSALTL | 15.44 | 10 | mmpL3 | Conserved transmembrane transport protein mmpL3 | A0A0H3M2S6 | HLA-A*02:01 | 3716.2 |
| IP: IVA12  Sample 5: + IFNγ / anti-IL-10 + HK-BCG | | | | | | | | |
| 1 | AQTGVYEDLLAAGVADPVK | 67.66 | 19 | groEL2 | 60 kDa chaperonin 2 | A1KFR2 | DRB1*01:01 | 235.24 |
| 2 | TGVYEDLLAAGVADPVK | 60.75 | 17 | groEL2 | 60 kDa chaperonin 2 | A1KFR2 | DRB1*01:01 | 131.35 |
| 3 | NGEEYLILSARDVLA | 60.72 | 15 | groS | 10 kDa chaperonin | A1KPA9 | DRB1*01:01 | 7.44 |
| 4 | DNQPSVQIQVYQGEREIAAHNKL | 57.14 | 23 | dnaK | Chaperone protein DnaK | A1KFH2 | DRB1_1501 | 373.31 |
| 5 | DTNYHYLVPEIGPS | 46.86 | 14 | metE | 5-methyltetrahydropteroyltriglutamate--homocysteine methyltransferase | A1KHS4 | DRB1*01:01 | 78.63 |
| 6 | DPFEDLGAQLVK | 45.44 | 12 | groL1 | 60 kDa chaperonin 1 | A1KPA8 | DRB1*01:01 | 82.84 |
| 7 | YLILSARDVLA | 35.95 | 11 | groS | 10 kDa chaperonin | A1KPA9 | HLA-A*02:01 | 1900 |
| 8 | LQDMAILTGGQVIS | 34.93 | 14 | groEL2 | 60 kDa chaperonin 2 | A1KFR2 | DRB1*01:01 | 62.12 |
| 9 | WDNGTTHD | 28.9 | 8 | ctpI | Probable cation-transporter atpase I ctpI | A0A0H3M0P5 | HLA-B*35:01 | 39648.2 |
| 10 | FRDEQGQDVLL | 21.4 | 11 | atpD | ATP synthase subunit beta | A1KI98 | HLA-A*02:01 | 30156.6 |
| 11 | FLKFIATEIFP | 18.69 | 11 | mmaA2 | Methoxy mycolic acid synthase 2 mmaA2 | A0A0H3M233 | HLA-A*02:01 | 5751.1 |
| 12 | IAGRFGLLD | 16.45 | 9 | mmpL9a | Probable conserved transmembrane transport protein mmpL9a | A0A0H3M636 | HLA-B*35:01 | 25361 |
| 13 | DDLLRPGI | 16.4 | 8 | subI | Probable sulfate-binding lipoprotein subI | A0A0H3MFF9 | HLA-A*02:01 | 40067.9 |
| 14 | SPPDNTIRAFRDEVLAA | 15.87 | 17 | cysA2 | Sulfurtransferase | A0A0H3MB67\|A0A0H3M8T4 | DQA1*01:01/DQB1*05:01 | 14.37 |
| 15 | GLTGIIMRATI | 15.23 | 11 | BCG_3852 | Putative oxidoreductase | A0A0H3MC09 | HLA-A*02:01 | 1168.6 |
| 16 | KLIENLRADT | 15.17 | 10 | mmpL12 | Probable conserved transmembrane transport protein mmpL12 | A0A0H3M689 | HLA-A*02:01 | 1653.3 |
| IP: IVA12  Sample 6: + IFNγ / anti-IL-10 + live BCG | | | | | | | | |
| 1 | AQTGVYEDLLAAGVADPVK | 76.47 | 19 | groEL2 | 60 kDa chaperonin 2 | A1KFR2 | DRB1*01:01 | 235.24 |
| 2 | NGEEYLILSARDVLA | 68.19 | 15 | groS | 10 kDa chaperonin | A1KPA9 | DRB1*01:01 | 7.44 |
| 3 | ANRAELKALIASNLLGQNT | 44.17 | 19 | PPE50 | PPE family protein | A0A0H3M8U4 | DRB1*01:01 | 18.45 |
| 4 | LQDMAILTGGQVIS | 39.74 | 14 | groEL2 | 60 kDa chaperonin 2 | A1KFR2 | DRB1*01:01 | 62.12 |
| 5 | YLILSARDVLA | 35.2 | 11 | groS | 10 kDa chaperonin | A1KPA9 | HLA-A*02:01 | 1900 |
| 6 | DTNYHYLVPEIGPS | 25.94 | 14 | metE | 5-methyltetrahydropteroyltriglutamate--homocysteine methyltransferase | A1KHS4 | DRB1*01:01 | 78.63 |
| 7 | NGEEYLILSARDVL | 25.25 | 14 | groS | 10 kDa chaperonin | A1KPA9 | DRB1*01:01 | 35.13 |
| 8 | DDLLRPGI | 23.71 | 8 | subI | Probable sulfate-binding lipoprotein subI | A0A0H3MFF9 | HLA-A*02:01 | 40067.9 |
| 9 | PGIPFGAA | 21.71 | 8 | BCG_0106 | Probable aminotransferase | A0A0H3M786 | HLA-B*35:01 | 37570.9 |
| 10 | GTTTYSSTAQHSA | 19.16 | 13 | BCG_2720 | Probable conserved transmembrane alanine and leucine rich protein | A0A0H3MD69 | DQA1*01:02/DQB1*06:02 | 1110.6 |
| 11 | TIAMLETAGLWG | 16.49 | 12 | BCG_1796 | Possible sulfite oxidase | A0A0H3MDW2 | DRB1*01:01 | 105.04 |
| 12 | LAIDVSDPRVI | 16.35 | 11 | BCG_3198 | Probable short-chain dehydrogenase/reductase | A0A0H3MHA8 | HLA-C*03:03 | 15192.9 |
| 13 | LRTLRDDPADAEVASHKLL | 15.73 | 19 | proS | Proline--tRNA ligase | A1KMI8 | DQA1*01:01/DQB1*05:01 | 4339.54 |
| 14 | WPAPYGRG | 15.47 | 8 | fadE34 | Probable acyl-CoA dehydrogenase fadE34 | A0A0H3MIH3 | HLA-B*35:01 | 23268.7 |
| 15 | GGAGGAGGWLIGQSGSTGGGGAGG | 15.25 | 24 | PE_PGRS48 | PE-PGRS family protein | A0A0H3M9S3 | DRB1*01:01 | 1008.12 |
| 16 | KPEDRYASAG | 15.01 | 10 | pknH | Probable transmembrane serine/threonine-protein kinase H pknH | A0A0H3M9Y5 | HLA-B*35:01 | 18808.2 |

**Supplementary Table 2:** Identified *MTB* peptides and corresponding proteins.

| **Number** | **Peptide** | **Peaks score**  **(-10lgP)** | **Length** | **Gene** | **Protein** | **Accession** | **Predicted THP-1 cell HLA allele**  **(best hit)** | **Predicted affinity (IC50)** |
| --- | --- | --- | --- | --- | --- | --- | --- | --- |
| IP: B721  Sample 2: + IFNγ / anti-IL-10 + live *MTB* | | | | | | | | |
| 1 | RAKARKAKAPK | 24.62 | 11 | Rv2164c | Probable conserved proline rich membrane protein | O06213 | HLA-C*03:03 | 38933.6 |
| 2 | TPHTEFEGQVYILSKDEGG | 24.54 | 19 | tuf | Elongation factor Tu | P9WNN1 | DRB1*01:01 | 2256.68 |
| 3 | ARAKARKAKAPK | 18.44 | 12 | Rv2164c | Probable conserved proline rich membrane protein | O06213 | DRB1*01:01 | 10576.08 |
| 4 | VGMPAPA | 18.3 | 7 | pssA | CDP-diacylglycerol--serine O-phosphatidyltransferase | P9WPG1 |  |  |
| 5 | ETRLDMSIPAP | 17.82 | 11 | dnaX | DNA polymerase III subunit gamma/tau | P9WNT9 | HLA-A*02:01 | 25762.6 |
| 6 | RLAIADDLAF | 17.38 | 10 | Rv2282c | Uncharacterized HTH-type transcriptional regulator | P9WMF3 | HLA-B*35:01 | 154 |
| 7 | TTYTLEYDG | 17.24 | 9 | PE1 | PE family protein PE1 | Q79G06 | HLA-B*35:01 | 18697.8 |
| 8 | AGTWVKAVAGIRLALSLGFRVRVA | 16.67 | 24 | moeX | Possible molybdopterin biosynthesis protein MoeX | O33183 | DRB1*01:01 | 36.15 |
| 9 | RPDRIVVGEVRGAEVVDLLAAL | 16.6 | 22 | Rv3659c | Putative conjugal transfer protein | P9WMT3 | DQA1*01:02/DQB1*06:02 | 266.43 |
| 10 | KAPAGGTGGAGGDGGAGGKG | 15.93 | 20 | PE_PGRS56 | PE-PGRS family protein PE_PGRS56 | Q6MWW7 | DQA1*01:02/DQB1*06:02 | 22863.62 |
| 11 | TVGPARATFR | 15.88 | 10 | helZ | Probable helicase HelZ | I6YCF3 | HLA-A*24:02 | 12867.8 |
| 12 | KLGAVAVPINTRLAAP | 15.81 | 16 | fadD13 | Long-chain-fatty-acid--CoA ligase FadD13 | P9WQ37 | DRB1*01:01 | 505.24 |
| 13 | VTPVKPGTPGEPTPITPVTPPVAP | 15.65 | 24 | espK | ESX-1 secretion-associated protein EspK | P9WJC1 | DRB1*01:01 | 3223.66 |
| 14 | APGAVDPAGLRAQLAQRLPGYLVP | 15.37 | 24 | nrp | Phenyloxazoline synthase MbtB | Q10896 | DRB1*01:01 | 35.82 |
| 15 | KPFELMELDLDGP | 15.31 | 13 | adhD | Putative alcohol dehydrogenase D | P9WQB9 | DQA1*01:01/DQB1*05:01 | 1156.37 |
| 16 | AAPKNDLSWHDCT | 15.04 | 13 | Rv2672 | Possible secreted protease | P71969 | DQA1*01:02/DQB1*06:02 | 29440.56 |
| IP: IVA12  Sample 2: + IFNγ / anti-IL-10 + live *MTB* | | | | | | | | |
| 1 | AQTGVYEDLLAAGVADPVK | 50.03 | 19 | groEL2 | Heat shock protein 65 | P9WPE7 | DRB1*01:01 | 235.24 |
| 2 | DAGGTYQPHPAEAVVE | 38.99 | 16 | fadB | 3-hydroxyacyl-CoA dehydrogenase | P9WNP7 | DRB1*01:01 | 804.57 |
| 3 | NGEEYLILSARDVLA | 31.38 | 15 | groS | Co-chaperonin GroES | I6YG32 | DRB1*01:01 | 7.44 |
| 4 | NGEEYLILSARDVL | 30.61 | 14 | groS | Co-chaperonin GroES | I6YG32 | DRB1*01:01 | 35.13 |
| 5 | DMAILTGGQVISEE | 27.16 | 14 | groEL2 | Heat shock protein 65 | P9WPE7 | DRB1*01:01 | 107.03 |
| 6 | GVDPLTAIRY | 25.37 | 10 | Rv3000 | Possible conserved transmembrane protein | I6X5Z8 | HLA-B*35:01 | 10684.4 |
| 7 | PVLVYLTIT | 24.54 | 9 | irtA | Mycobactin import ATP-binding/permease protein IrtA | P9WQJ9 | HLA-A*02:01 | 19227.6 |
| 8 | DMAILTGGQVISEEVG | 22.7 | 16 | groEL2 | Heat shock protein 65 | P9WPE7 | DRB1*01:01 | 112.76 |
| 9 | KSKGAKGKSGKK | 21.3 | 12 | ffh | Signal recognition particle protein | P9WGD7 | DRB5*01:01 | 11454.34 |
| 10 | KAAPRVAKAAKAAPKK | 20.5 | 16 | rpsQ | 30S ribosomal protein S17 | P9WH51 | DRB5*01:01 | 65.46 |
| 11 | ESLQEVARAAGLQ | 19.93 | 13 | fadD9 | Carboxylic acid reductase | Q50631 | DQA1*01:02/DQB1*06:02 | 2121.92 |
| 12 | GGLGNRGGAGGAGGAGGVGG | 18.72 | 20 | PE_PGRS21 | PE-PGRS family protein PE_PGRS21 | Q79FT0 | DQA1*01:02/DQB1*06:02 | 13019.99 |
| 13 | EVTRELQAL | 18.66 | 9 | pks17 | Probable polyketide synthase Pks17 | O06585 | HLA-C*03:03 | 6089.7 |
| 14 | YSFVVLLIT | 17.47 | 9 | qcrB | Cytochrome bc1 complex cytochrome b subunit | P9WP37 | HLA-A*02:01 | 3383.6 |
| 15 | QALTAGAGAYAFAEAA | 17.11 | 16 | PE_PGRS53 | PE-PGRS family protein PE_PGRS53 | Q6MWW9 | DRB1*01:01 | 56.83 |
| 16 | PMFEAPGRITELITSFIEE | 16.95 | 19 | Rv3338 | AB hydrolase-1 domain-containing protein | O53388 | DQA1*01:02/DQB1*06:02 | 1252.4 |
| 17 | GTGGAGGAGGLGGHGGAGGLLIGNG | 16.88 | 25 | PE_PGRS48 | PE-PGRS family protein | Q6MX26 | DQA1*01:02/DQB1*06:02 | 7442.62 |
| 18 | WPQPAQP | 16.87 | 7 | Rv0926c | DAP_DH_C domain-containing protein | I6WZS8 |  |  |
| 19 | AIEKTFAAI | 16.52 | 9 | Rv1488 | Uncharacterized protein | P9WPR9 | HLA-A*02:01 | 5314.8 |
| 20 | WVQEYLEATARLDAL | 16.5 | 15 | Rv1894c | NMO domain-containing protein | O07738 | DRB1*01:01 | 35.22 |
| 21 | NAVVVKPVPPSAVVVGVPGQVIGQ | 16.33 | 24 | cysE | Serine acetyltransferase | P95231 | DRB1*01:01 | 190.54 |
| 22 | PLKQIAFNSGLEPGVVAEKVRNLP | 16.3 | 24 | groEL2 | Heat shock protein 65 | P9WPE7 | DRB1*01:01 | 390.58 |
| 23 | KTPVSRMRPTRCCCPAAP | 16.13 | 18 | Rv2251 | Possible flavoprotein | L0TBR2 | DRB1*01:01 | 3888.08 |
| 24 | VPAISLPLAQSADGMPVGMML | 15.75 | 21 | amiC | Putative amidase AmiC | I6Y4D2 | DQA1*01:02/DQB1*06:02 | 1033.05 |
| 25 | KPAEEGDVVGASEPDAK | 15.74 | 17 | rplQ | 50S ribosomal protein L17 | P9WHD3 | DQA1*01:02/DQB1*06:02 | 3368.72 |
| 26 | KAVSKAARAK | 15.7 | 10 | secE | Protein translocase subunit SecE | P9WGN7 | HLA-B*35:01 | 36682 |
| 27 | TLISAGAVEY | 15.67 | 10 | mltG | Endolytic murein transglycosylase | I6XEK6 | HLA-B*35:01 | 2455.7 |
| 28 | FGLTQNVYLVFAAT | 15.58 | 14 | Rv2723 | Uncharacterized membrane protein | P9WG93 | DPA1*01:03/DPB1*02:01 | 201.4 |
| 29 | GVGGAGGQGGLLFGDGGNGGA | 15.57 | 21 | PE_PGRS58 | PE-PGRS family protein PE_PGRS58 | I6XHM5 | DQA1*01:02/DQB1*06:02 | 7706.81 |
| 30 | LAQQGFFKG | 15.39 | 9 | ppiB | Probable peptidyl-prolyl cis-trans isomerase B | P9WHW1 | HLA-B*35:01 | 5922 |
| 31 | GYDFLPGTTVN | 15.28 | 11 | fadA3 | Probable beta-ketoacyl CoA thiolase FadA3 | O53422 | HLA-A*02:01 | 30835.6 |
| 32 | FFKAYVVN | 15.22 | 8 | mce3C | Mce-family protein Mce3C | O53969 | HLA-A*02:01 | 32843.7 |
| 33 | EDALWRWMTAAALVRPRGAG | 15.13 | 20 | priA | Probable primosomal protein N | P9WMQ9 | DRB1*01:01 | 5.81 |
| 34 | LTILLACSAIDTSSAAALL | 15.09 | 19 | Rv0235c | Probable conserved transmembrane protein | P96418 | DQA1*01:02/DQB1*06:02 | 322.27 |
| IP: W6/32  Sample 2: + IFNγ / anti-IL-10 + live *MTB* | | | | | | | | |
| 1 | FIRITDETL | 25.85 | 9 | Rv0306 | Oxidoreductase | O07233 | HLA-C*03:03 | 2793.8 |
| 2 | PRDWHQVTVVSSPSWFSDY | 24.33 | 19 | mycP2 | Mycosin-2 | O05458 | DQA1*01:02/DQB1*06:02 | 353.62 |
| 3 | SHGSAYPSTEQT | 23.59 | 12 | lprM | Possible Mce-family lipoprotein LprM | O53971 | DQA1*01:02/DQB1*06:02 | 16811.26 |
| 4 | AAKKAPARK | 20.38 | 9 | hbs | Possible histone-like protein Hns | I6YHB0 | HLA-C*03:03 | 6354.1 |
| 5 | TLSGLAPATL | 20.12 | 10 | Rv3233c | Possible triacylglycerol synthase | O05878 | HLA-A*02:01 | 527.1 |
| 6 | VIHVVADASAL | 20.11 | 11 | Rv2209 | Uncharacterized protein | P9WLI3 | HLA-A*02:01 | 19119.3 |
| 7 | RVGIDAEAGPT | 19.71 | 11 | hisD | Histidinol dehydrogenase | P9WNW9 | HLA-A*02:01 | 37508.7 |
| 8 | ALAIGAI | 19.01 | 7 | Rv0426c | Possible transmembrane protein | P96272 |  |  |
| 9 | SHGSAYPSTEQ | 18.71 | 11 | lprM | Possible Mce-family lipoprotein LprM | O53971 | HLA-B*35:01 | 38683 |
| 10 | IEIGAGV | 18.4 | 7 | Rv0161 | Possible oxidoreductase | O07406 |  |  |
| 11 | QLMLRPVL | 18.07 | 8 | Rv0998 | Acetyltransferase Pat | O05581 | HLA-A*02:01 | 5240.6 |
| 12 | TPKKMIAA | 17.83 | 8 | rplD | 50S ribosomal protein L4 | P9WH85 | HLA-B*35:01 | 30324.8 |
| 13 | IAQKHATI | 17.68 | 8 | Rv2209 | Uncharacterized protein | P9WLI3 | HLA-C*03:03 | 5418.1 |
| 14 | LVTDDLIEY | 17.4 | 9 | Rv3359 | Possible oxidoreductase | O50388 | HLA-B*35:01 | 12 |
| 15 | VHADAAPA | 17.24 | 8 | Rv2642 | mannose-6-phosphate isomerase | P71941 | HLA-A*02:01 | 32176.8 |
| 16 | LAARLPLAY | 17.2 | 9 | Rv1204c | AAA domain-containing protein | O05305 | HLA-B*35:01 | 4.7 |
| 17 | VLAVVIIGVL | 17.06 | 10 | Rv0544c | Possible conserved transmembrane protein | O06410 | HLA-A*02:01 | 219.1 |
| 18 | TLADGTVLGYD | 17.01 | 11 | Rv0688 | Ferredoxin reductase | P95034 | HLA-B*35:01 | 21972.8 |
| 19 | TAQNVIDASV | 16.91 | 10 | Rv3365c | Conserved protein | Q93IG6 | HLA-A*02:01 | 5064.3 |
| 20 | IAINLRGAW | 16.72 | 9 | Rv1941 | Probable short-chain type dehydrogenase/reductase | I6XZC4 | HLA-C*03:03 | 1340.6 |
| 21 | AGKVLRI | 16.61 | 7 | lppZ | Probable conserved lipoprotein LppZ | I6Y293 |  |  |
| 22 | SDAAHDMVSSCA | 16.6 | 12 | Rv2488c | Probable transcriptional regulatory protein | O53213 | DQA1*01:02/DQB1*06:02 | 2485.04 |
| 23 | LENVRPLA | 16.57 | 8 | Rv3239c | Probable conserved transmembrane transport protein | O05884 | HLA-A*02:01 | 25073.1 |
| 24 | AVAAPTG | 16.52 | 7 | MmpL13b | Probable conserved transmembrane transport protein MmpL13b |  |  |  |
| 25 | MIGAGIFAAL | 16.34 | 10 | Rv1999c | Uncharacterized transporter | P9WQM3 | HLA-A*02:01 | 201.9 |
| 26 | TLAAFLWAL | 16.32 | 9 | lhr | Probable ATP-dependent helicase Lhr | P96901 | HLA-A*02:01 | 4.3 |
| 27 | LAGELKAAGDDY | 15.95 | 12 | Rv1260 | Uncharacterized protein | P9WM51 | DQA1*01:02/DQB1*06:02 | 7446.48 |
| 28 | TTDLLYEVD | 15.82 | 9 | Rv3472 | Conserved protein | I6YG83 | HLA-B*35:01 | 31914 |
| 29 | TVELDEPLV | 15.8 | 9 | dlaT | Dihydrolipoyllysine-residue acetyltransferase | P9WIS7 | HLA-A*02:01 | 8355.7 |
| 30 | LGGAGVFAV | 15.59 | 9 | SugI | Probable sugar-transport integral membrane protein SugI | L0TDU1 | HLA-A*02:01 | 4662.3 |
| 31 | IGPTHTL | 15.5 | 7 | Rv3776 | Uncharacterized protein | P72042 |  |  |
| 32 | LFAGDLLFI | 15.49 | 9 | Rv0939 | Possible bifunctional enzyme | O86346 | HLA-A*24:02 | 1382.5 |
| 33 | TVAFPKPSV | 15.46 | 9 | Rv1320c | Uncharacterized protein | P9WQ29 | HLA-C*03:03 | 6039 |
| 34 | TVHGKCGSLTL | 15.44 | 11 | manA | mannose-6-phosphate isomerase | O05898 | HLA-A*02:01 | 20923.2 |
| 35 | IAVGKLAL | 15.43 | 8 | mez | Putative malate oxidoreductase | P9WK25 | HLA-C*03:03 | 1208.1 |
| 36 | ELLSRAHQL | 15.28 | 9 | Rv0024 | Secreted protein P60-related protein | O06546 | HLA-A*02:01 | 3406.6 |
| 37 | GFAEIAL | 15.13 | 7 | ppsD | Phenolphthiocerol/phthiocerol polyketide synthase subunit D | P9WQE3 |  |  |

**Supplementary Table 3:** Immunopeptidomics and immunoinformatics information of each selected antigens.

| **No** | **Antigen** | **Peptide** | **Source of peptide** | **Peaks score**  **-10lgP** | **Peptide**  **Length** | **Antigen**  **Length** | **Human MHC Coverage %** | **Mouse MHC Coverage %** |
| --- | --- | --- | --- | --- | --- | --- | --- | --- |
| 1 | **groEL2** | AQTGVYEDLLAAGVADPVK | BCG live infection (sample 3/6) | 76.47 | 19 | 540 | 96.3 | 62.5 |
|  |  | AQTGVYEDLLAAGVADPVK | *MTB* live infection | 50.03 |  |  |  |  |
|  |  | AQTGVYEDLLAAGVADPVK | BCG live infection (Bettencourt et al, 2020) | 35.11 |  |  |  |  |
|  |  | AQTGVYEDLLAAGVADPVK | BCG HK infection (sample 2/5) | 67.66 |  |  |  |  |
|  |  | DMAILTGGQVISEE | *MTB* live infection | 27.16 | 14 |  |  |  |
|  |  |  |  |  |  |  |  |  |
|  |  | DMAILTGGQVISEEVG | *MTB* live infection | 22.7 | 16 |  |  |  |
|  |  | DPYEKIGAELVK | BCG HK infection (Bettencourt et al, 2020) | 68.08 | 12 |  |  |  |
|  |  |  |  |  |  |  |  |  |
|  |  |  |  |  |  |  |  |  |
|  |  | DPYEKIGAELVKEVA | BCG HK infection (Bettencourt et al, 2020) | 46.93 | 15 |  |  |  |
|  |  |  |  |  |  |  |  |  |
|  |  |  |  |  |  |  |  |  |
|  |  |  |  |  |  |  |  |  |
|  |  |  |  |  |  |  |  |  |
|  |  | DPYEKIGAELVKEVAK | BCG HK infection (Bettencourt et al, 2020) | 49.14 | 16 |  |  |  |
|  |  |  |  |  |  |  |  |  |
|  |  |  |  |  |  |  |  |  |
|  |  |  |  |  |  |  |  |  |
|  |  |  |  |  |  |  |  |  |
|  |  | EDPYEKIGAELVKE | BCG HK infection (Bettencourt et al, 2020) | 45.4 | 14 |  |  |  |
|  |  |  |  |  |  |  |  |  |
|  |  |  |  |  |  |  |  |  |
|  |  |  |  |  |  |  |  |  |
|  |  |  |  |  |  |  |  |  |
|  |  |  |  |  |  |  |  |  |
|  |  | LEDPYEKIGAELVKE | BCG HK infection (Bettencourt et al, 2020) | 30.75 | 15 |  |  |  |
|  |  |  |  |  |  |  |  |  |
|  |  |  |  |  |  |  |  |  |
|  |  |  |  |  |  |  |  |  |
|  |  |  |  |  |  |  |  |  |
|  |  |  |  |  |  |  |  |  |
|  |  | LQDMAILTGGQVIS | BCG live infection (sample 3/6) | 39.74 | 14 |  |  |  |
|  |  |  |  |  |  |  |  |  |
|  |  | LQDMAILTGGQVIS | BCG HK infection (sample 2/5) | 34.93 |  |  |  |  |
|  |  | TGVYEDLLAAGVADPVK | BCG HK infection (sample 2/5) | 60.75 | 17 |  |  |  |
| 2 | **groS** | NGEEYLILSARDVL | BCG live infection (sample 3/6) | 25.25 | 14 | 100 | 88.8 | 12.5 |
|  |  | NGEEYLILSARDVL | *MTB* live infection | 30.61 |  |  |  |  |
|  |  | NGEEYLILSARDVLA | BCG live infection (sample 3/6) | 68.19 | 15 |  |  |  |
|  |  | NGEEYLILSARDVLA | BCG live infection (Bettencourt et al, 2020) | 68.22 |  |  |  |  |
|  |  | NGEEYLILSARDVLA | *MTB* live infection | 31.38 |  |  |  |  |
|  |  | NGEEYLILSARDVLA | BCG HK infection (sample 2/5) | 60.72 |  |  |  |  |
|  |  | YLILSARDVLA | BCG live infection (sample 3/6) | 35.2 | 11 |  |  |  |
|  |  | YLILSARDVLA | BCG live infection (Bettencourt et al, 2020) | 41.23 |  |  |  |  |
|  |  | YLILSARDVLA | BCG HK infection (sample 2/5) | 35.95 |  |  |  |  |
| 3 | **dnaK** | DNQPSVQIQVYQGEREIAAHNKL | BCG HK infection (sample 2/5) | 57.14 | 23 | 625 | 100 | 87.5 |
|  |  |  |  |  |  |  |  |  |
| 4 | **metE** | DTNYHYLVPEIGPS | BCG HK infection (sample 2/5) | 46.86 | 14 | 759 | 100 | 100 |
|  |  | DTNYHYLVPEIGPS | BCG live infection (sample 3/6) | 25.94 |  |  |  |  |
|  |  | DTNYHYLVPEIGPS | BCG HK infection (Bettencourt et al, 2020) | 31.61 |  |  |  |  |
| 5 | **groL1** | DPFEDLGAQLVK | BCG HK infection (sample 2/5) | 45.44 | 12 | 539 | 100 | 87.5 |
|  |  |  |  |  |  |  |  |  |
| 6 | **clpP1** | ADIAIQAEQFAVIKK | BCG HK infection (sample 2/5) | 45.19 | 15 | 200 | 88.9 | 62.5 |
|  |  | ADIAIQAEQFAVIKK | BCG live infection (sample 3/6) | 31.37 |  |  |  |  |
| 7 | **PPE50** | ANRAELKALIASNLLGQNT | BCG live infection (sample 3/6) | 44.17 | 19 | 381 | 92.6 | 100 |
|  |  | ANRAELKALIASNLLGQNT | BCG live infection (Bettencourt et al, 2020) | 39.94 |  |  |  |  |
| 8 | **fadB** | DAGGTYQPHPAEAVVE | *MTB* live infection | 38.99 | 16 | 720 | 100 | 100 |
|  |  |  |  |  |  |  |  |  |
|  |  |  |  |  |  |  |  |  |
|  |  |  |  |  |  |  |  |  |
|  |  |  |  |  |  |  |  |  |
| 9 | **ctpI** | WDNGTTHD | BCG HK infection (sample 2/5) | 32.83 | 8 | 1,625 | 100 | 100 |
| 10 | **BCG_3870c** | LAASLLSRV | BCG HK infection (Bettencourt et al, 2020) | 28.16 | 9 | 637 | 100 | 100 |
|  |  | TVAIQNKAI | BCG live infection (sample 3/6) | 19.57 | 9 |  |  |  |
| 11 | **Rv0306 (BCG_0346)** | FIRITDETL | *MTB* live infection | 25.85 | 9 | 223 | 81.5 | 87.5 |
| 12 | **Rv3000 (BCG_3022)** | GVDPLTAIRY | *MTB* live infection | 25.37 | 10 | 219 | 85.2 | 75 |
| 13 | **fadE17** | KHRGLSFL | BCG live infection (sample 3/6) | 25.07 | 8 | 409 | 100 | 87.5 |
|  |  | KHRGLSFL | BCG HK infection (sample 2/5) | 21.91 |  |  |  |  |
| 14 | **mmpL12** | AGCTLLIR | BCG live infection (Bettencourt et al, 2020) | 24.98 | 8 | 1,107 | 100 | 100 |
|  |  | KALMSIGKVITASAAT | BCG live infection (sample 3/6) | 16.7 | 16 |  |  |  |
|  |  | KLIENLRADT | BCG HK infection (sample 2/5) | 15.17 | 10 |  |  |  |
| 15 | **Rv2164c (BCG_2181c)** | RAKARKAKAPK | *MTB* live infection | 24.62 | 11 | 384 | 70.4 | 75 |
| 16 | **tuf** | TPHTEFEGQVYILSKDEGG | *MTB* live infection | 24.54 | 19 | 396 | 100 | 75 |
|  |  |  |  |  |  |  |  |  |
| 17 | **irtA (BCG_1410)** | PVLVYLTIT | *MTB* live infection | 24.54 | 9 | 859 | 100 | 100 |
| 18 | **mycP2 (BCG_3941c)** | PRDWHQVTVVSSPSWFSDY | *MTB* live infection | 24.33 | 19 | 550 | 100 | 87.5 |
| 19 | **subI** | DDLLRPGI | BCG live infection (sample 3/6) | 23.71 | 8 | 356 | 100 | 87.5 |
|  |  | DDLLRPGI | BCG HK infection (sample 2/5) | 16.4 |  |  |  |  |
| 20 | **lprM (Rv1970)** | SHGSAYPSTEQT | *MTB* live infection | 23.59 | 12 | 377 | 85.2 | 87.5 |
| 21 | **hbhA** | KKAAPAKKAAPAK | BCG HK infection (sample 2/5) | 23.04 | 13 | 199 | 74 | 25 |
|  |  | KKAAAKKAPAKKAAAKK | BCG live infection (Bettencourt et al, 2020) | 28.56 | 17 |  |  |  |
|  |  | KAPAKKAAAKKVTQK | BCG HK infection (Bettencourt et al, 2020) | 24.92 | 15 |  |  |  |
|  |  | KAPAKKAAAK | BCG HK infection (Bettencourt et al, 2020) | 21.26 | 10 |  |  |  |
|  |  | KAAPAKKAAPAK | BCG live infection (sample 3/6) | 22.72 | 12 |  |  |  |
|  |  | KAAPAKKAAPAK | BCG HK infection (sample 2/5) | 18.16 |  |  |  |  |
|  |  | AKKAAPAKKAAPAKKAAAKK | BCG live infection (Bettencourt et al, 2020) | 17.73 | 20 |  |  |  |
|  |  | AAAKKAPAKKAAAKK | BCG live infection (Bettencourt et al, 2020) | 17.24 | 15 |  |  |  |
| 22 | **BCG_2418** | TVRCVSGTVEL | BCG HK infection (sample 2/5) | 23 | 11 | 642 | 100 | 100 |
|  |  | TVRCVSGTVEL | BCG live infection (sample 3/6) | 17.4 |  |  |  |  |
| 23 | **BCG_0106** | PGIPFGAA | BCG live infection (sample 3/6) | 21.71 | 8 | 390 | 96.3 | 100 |
| 24 | **atpD** | FRDEQGQDVLL | BCG HK infection (sample 2/5) | 21.4 | 11 | 486 | 96.3 | 100 |
| 25 | **cysA2** | EVDEDTSAYD | BCG HK infection (sample 2/5) | 21.33 | 10 | 277 | 96.3 | 75 |
| 26 | **ffh (BCG_2937c)** | KSKGAKGKSGKK | *MTB* live infection | 21.3 | 12 | 525 | 92.6 | 87.5 |
| 27 | **rplV** | KAPAKKAPAKASETSAAKGGS | BCG live infection (Bettencourt et al, 2020) | 21.12 | 21 | 197 | 100 | 50 |
|  |  | KKAPAKASET | BCG HK infection (sample 2/5) | 16.93 | 10 |  |  |  |
|  |  | PAKKAAAKAPA | BCG live infection (Bettencourt et al, 2020) | 16.31 | 11 |  |  |  |
| 28 | **rpsQ (BCG_0760)** | KAAPRVAKAAKAAPKK | *MTB* live infection | 20.5 | 16 | 136 | 74 | 50 |
|  |  |  |  |  |  |  |  |  |
|  |  |  |  |  |  |  |  |  |
|  |  |  |  |  |  |  |  |  |
| 29 | **hbs (BCG_3915)** | AAKKAPARK | *MTB* live infection | 20.38 | 9 | 134 | 37 | 50 |
| 30 | **Rv3233c (BCG_3263c)** | TLSGLAPATL | *MTB* live infection | 20.12 | 10 | 469 | 100 | 100 |
|  |  |  |  |  |  |  |  |  |
| 31 | **Rv2209 (BCG_2119)** | VIHVVADASAL | *MTB* live infection | 20.11 | 11 | 550 | 96.3 | 100 |
| 32 | **hisD** | KDVSGHVITL | BCG HK infection (sample 2/5) | 20.04 | 10 | 438 | 92.6 | 100 |
|  |  | RVGIDAEAGPT | BCG live infection (Bettencourt et al, 2020) | 19.9 | 11 |  |  |  |
|  |  | RVGIDAEAGPT | *MTB* live infection | 19.71 |  |  |  |  |
| 33 | **fadE34** | RLGVDLAEV | BCG live infection (Bettencourt et al, 2020) | 19.13 | 9 | 711 | 100 | 100 |
|  |  | WPAPYGRG | BCG live infection (sample 3/6) | 15.47 | 8 |  |  |  |
| 34 | **PPE8** | EINSARLYV | BCG HK infection (Bettencourt et al, 2020) | 18.25 | 9 | 3,507 | 100 | 100 |
|  |  | ITNVSIPAI | BCG HK infection (Bettencourt et al, 2020) | 16.41 | 9 |  |  |  |
|  |  | TGFYNTGDVNT | BCG HK infection (sample 2/5) | 16.08 | 11 |  |  |  |
|  |  | GFYNTGSYNTGGFN | BCG live infection (sample 3/6) | 16.03 | 14 |  |  |  |
| 35 | **PPE55a** | GDYEGLWGL | BCG live infection (Bettencourt et al, 2020) | 17.95 | 9 | 2,096 | 89 | 87.5 |
|  |  | DFDGAADAGFTAPATTL | BCG live infection (sample 3/6) | 15.04 | 17 |  |  |  |
| 36 | **pks12** | VTVNATPD | BCG live infection (Bettencourt et al, 2020) | 16.06 | 8 | 4,151 | 100 | 100 |
|  |  | LATLFGDGVLRPLPV | BCG HK infection (sample 2/5) | 16.09 | 15 |  |  |  |
| 37 | **mmpL3** | RTLFRVPF | BCG HK infection (Bettencourt et al, 2020) | 15.38 | 8 | 944 | 100 | 100 |
|  |  | KATLMSALTL | BCG live infection (sample 3/6) | 15.44 | 10 |  |  |  |
| 38 | **hupB** | PAKKAAAKRPATKAPAKKATARR | BCG HK infection (Bettencourt et al, 2020) | 15.36 | 23 | 205 | 74 | 37.5 |
|  |  | KAPAKKAATKAPAKK | BCG live infection (sample 3/6) | 18.28 | 15 |  |  |  |
| 39 | **Rv0426c (BCG_0465c)** | ALAIGAI | *MTB* live infection | 19.01 | 7 | 147 | 33.3 | 12.5 |
|  |  | PVKAKLAPVP | BCG HK infection (Bettencourt et al, 2020) | 17.37 | 10 |  |  |  |
| 40 | **Rv2164c (BCG_2181c)** | ARAKARKAKAPK | *MTB* live infection | 18.44 | 12 | 384 | 70.4 | 75 |
|  |  | KPADGVPPPPLNTKLPEDPPP | BCG HK infection (sample 2/5) | 15.87 | 21 |  |  |  |
| 41 | **Rv3239c (BCG_3268c)** | LENVRPLA | *MTB* live infection | 16.57 | 8 | 1048 | 100 | 100 |
|  |  | KPNPIGVGLME | BCG HK infection (Bettencourt et al, 2020) | 16.32 | 11 |  |  |  |
| 42 | **Rv3776 (BCG_3838)** | IGPTHTL | *MTB* live infection | 15.5 | 7 | 519 | 92.6 | 100 |
|  |  | APLLAELIRGGAALSRVRHPGD | BCG HK infection (Bettencourt et al, 2020) | 16.23 | 22 |  |  |  |
| 43 | **nrp** | APGAVDPAGLRAQLAQRLPGYLVP | *MTB* live infection | 15.37 | 24 | 2,512 | 100 | 100 |
|  |  |  |  |  |  |  |  |  |
|  |  |  |  |  |  |  |  |  |
|  |  | KPLVRHTVHT | BCG live infection (sample 3/6) | 19.84 | 10 |  |  |  |
| 44 | **PE_PGRS48** | TGGAGGAGGLGGHGGAGGLLIGNG | *MTB* live infection | 16.88 | 25 | 597 | 59.3 | 75 |
|  |  | GGAGGAGGWLIGQSGSTGGGGAGG | BCG live infection (sample 3/6) | 15.25 | 24 |  |  |  |
| 45 | **PE_PGRS53** | QALTAGAGAYAFAEAA | *MTB* live infection | 17.11 | 16 | 1,372 | 85.2 | 75 |
|  |  |  |  |  |  |  |  |  |
|  |  | GTGGNASATGT | BCG HK infection (Bettencourt et al, 2020) | 19.84 | 11 |  |  |  |
| 46 | **PE1** | TTYTLEYDG | *MTB* live infection | 17.24 | 9 | 588 | 100 | 100 |
|  |  | TTYTLEYDG | BCG HK infection (sample 2/5) | 15.96 | 9 |  |  |  |
| 47 | **ppsD** | GFAEIAL | *MTB* live infection | 15.13 | 7 | 1,827 | 100 | 100 |
|  |  | GPAFAALS | BCG live infection (Bettencourt et al, 2020) | 16.51 | 8 |  |  |  |
